# Inhaled Nanomedicines for Lung Diseases and Injuries: Pharmacological Advances and Clinical Translation

**DOI:** 10.1002/mco2.71047

**Published:** 2026-11-04

**Authors:** Kajal Kamra, Sonal Gaur, Jaswinder Singh

**Affiliations:** ^1^ Department of Pharmaceutical Sciences University of Nebraska Medical Center Omaha Nebraska USA; ^2^ Department of Ophthalmology Medical University of South Carolina Charleston South Carolina USA; ^3^ Department of Emergency Medicine University of Nebraska Medical Center Omaha Nebraska USA

**Keywords:** alveolar regeneration, inhaled nanomedicine, lipid nanoparticles, precision pulmonary medicine, pulmonary drug delivery, respiratory gene therapy

## Abstract

Respiratory diseases remain major causes of morbidity and mortality; however, inadequate pulmonary drug exposure and systemic toxicity continue to limit treatment efficacy. Inhaled nanomedicines offer opportunities for localized, sustained, and targeted therapy. This review examines how disease biology informs pulmonary delivery design across pulmonary fibrosis, lung cancer, chronic obstructive pulmonary disease, pulmonary infections, and acute lung injury/acute respiratory distress syndrome. It outlines anatomical and biological barriers and compares liposomes, lipid nanoparticles, polymeric and hybrid systems, biomimetic carriers, and stimuli‐responsive formulations. Particular attention is given to matching therapeutic cargo and carrier properties to fibroblasts, macrophages, tumor cells, and alveolar type II cells. Emerging approaches include mRNA delivery, siRNA‐mediated gene silencing, antisense oligonucleotides, and CRISPR‐based editing, alongside alveolar regeneration and biomarker‐guided treatment. These strategies are evaluated in relation to their delivery requirements and stage of development. The review also addresses aerosol‐device compatibility, manufacturing, repeated‐dose safety, regulatory considerations, and clinical outcomes. By connecting delivery mechanisms with disease‐specific applications and clinical evidence, this review identifies both therapeutic opportunities and barriers to translation. This integrated perspective can guide the development of inhaled nanomedicines with more reproducible pulmonary delivery, improved treatment efficacy, and acceptable safety.

## Graphical abstract

Graphical overview of bioinspired regenerative platforms designed to remodel the diseased lung microenvironment. These systems integrate gene addition, siRNA‐mediated transcript silencing, CRISPR‐based modulation, and base editing approaches to promote alveolar repair, AT2‐to‐AT1 regeneration, resolution of inflammation, and restoration of vascular–epithelial integrity.

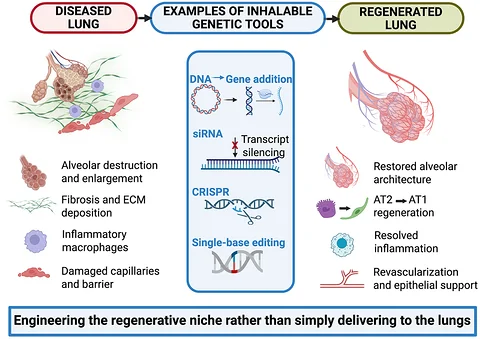

## 1 Introduction

Respiratory diseases remain a major global cause of death, hospital admission, and long‐term health care use. Chronic obstructive pulmonary disease (COPD), lower respiratory infections, tuberculosis, and lung cancer continue to rank among the leading causes of mortality worldwide [1]. Despite important advances in diagnosis and treatment, many respiratory disorders remain difficult to manage because therapeutic success depends not only on drug potency but also on achieving adequate and sustained drug exposure within complex pulmonary microenvironments. COPD is characterized by persistent airflow limitation, chronic inflammation, mucus hypersecretion, and structural remodeling of the airways and alveoli [2]. Interstitial lung disease, such as idiopathic pulmonary fibrosis (IPF), adds another layer of complexity, in which chronic epithelial injury, aberrant repair, and irreversible scarring of the gas exchange surface [3]. Lung cancer remains the leading cause of cancer‐related mortality worldwide, and despite substantial progress in targeted therapy and immunotherapy, many patients are still diagnosed with advanced stages [4]. Acute lung injury (ALI), acute respiratory distress syndrome (ARDS), and severe pulmonary infections further increase this burden by damaging the alveolar–capillary barrier and creating urgent therapeutic needs [5, 6, 7].

Current pulmonary pharmacotherapy has improved symptom control in many respiratory disorders, but it rarely solves the central delivery problem [8, 9]. Bronchodilators, corticosteroids, antibiotics, antifibrotics, and anticancer drugs often act in a broad and poorly targeted manner [8, 10]. This is particularly important because diseased lung tissue is rarely uniform. Fibrotic lesions, infected alveoli, inflamed airways, tumor nodules, and injured endothelium may each require distinct levels and patterns of drug exposure for optimal therapeutic effect [9]. Conventional inhaled drugs may deposit unevenly across the airways and can be cleared rapidly from the mucus or epithelial lining fluid turnover [8]. Systemically administered drugs can reach the lungs, but they also expose healthy tissues to off‐target toxicity. This limitation is especially relevant for chemotherapeutics, high‐dose corticosteroids, certain antimicrobials, and newer biologic agents [11, 12, 13].

Maintaining effective drug concentrations within diseased lung regions remains challenging, particularly in chronic respiratory disorders that require prolonged treatment. Short residence time in the lung can lead to fluctuating drug levels and weaker long‐term adherence. In infections and cancer, subtherapeutic exposure may also support resistant microbial or tumor cell populations [5, 14]. For chronic diseases such as COPD and pulmonary fibrosis, the challenge is even more fundamental. Many available treatments reduce symptoms or slow progression, but they do not fully reverse structural remodeling, restore damaged alveoli, or rebuild normal lung architecture [15, 16]. These limitations suggest that improving pulmonary drug delivery may be as important as developing new therapeutic molecules. Rather than simply increasing drug dose, nanomedicine seeks to improve where, when, and how therapeutics interact with diseased lung tissue. Nanomedicine offers a way to redesign pulmonary pharmacology around the biology of the diseased lung. Inhaled nanoparticles, liposomes, lipid nanoparticles, polymeric carriers, and hybrid systems can be engineered to improve drug stability, local retention, and controlled release within the respiratory tract [3]. Many promising therapies fail not because their biological targets lack relevance, but because adequate concentration cannot be maintained at the desired site of action for a sufficient duration. Nano‐enabled carriers can protect labile therapeutics from degradation, improve the apparent solubility of poorly water‐soluble compounds, and reduce premature systemic absorption [5].

A major advantage of nanomedicine is its potential to improve spatial and cellular precision within the lung. Aerodynamic properties primarily determine regional deposition, whereas nanocarrier size, surface chemistry, and mucus interactions become especially important after deposition by influencing retention, transport, and cellular access [17, 18, 19]. Further functionalization with peptides, antibodies, sugars, or other ligands may help nanoparticles interact more selectively with tumor cells, activated fibroblasts, infected macrophages, inflamed endothelium, or epithelial targets [20]. These challenges are particularly important for potent cargos such as nucleic acids, immunomodulators, antifibrotic agents, antimicrobials, and chemotherapeutics [10, 20].

Recent reviews have examined biological barriers, nanocarrier design, aerosol engineering, disease‐relevant target cells, and inhaled RNA delivery from complementary perspectives (Table S1). This review brings these elements together by connecting disease biology and target‐cell selection with therapeutic cargo, carrier design, and clinical evidence. The aim is to explain how these relationships inform disease‐specific treatment strategies and their translation into clinical practice. The review first examines pulmonary delivery barriers and nanomedicine platforms. It then discusses their applications in pulmonary fibrosis, lung cancer, COPD/emphysema, pulmonary infections, and ALI/ARDS. Subsequent sections compare nucleic acid therapies according to their mechanisms and delivery requirements, followed by clinical translation and regulatory considerations. Finally, the review considers precision treatment, regenerative approaches, and combination strategies before outlining the priorities for future development. Throughout, established clinical applications are distinguished from emerging preclinical approaches to clarify both therapeutic opportunities and remaining evidence gaps.

## 2 Biological and Pharmacological Barriers to Effective Pulmonary Drug Delivery

The lung offers a unique opportunity for local drug delivery, allowing therapeutics to be administered directly to diseased tissue while minimizing systemic exposure. However, efficient pulmonary delivery remains far from straightforward. Following inhalation, particles encounter a series of anatomical, physicochemical, and biological barriers that influence where they deposit, how long they remain in the lung, and whether they ultimately reach their intended cellular targets (Table 1; Table S2; Figure 1). These challenges become even more pronounced in diseased lungs, where structural remodeling, inflammation, fibrosis, infection, or tumor growth can substantially alter the pulmonary microenvironment.

**TABLE 1:** Biological barriers to inhaled therapeutics and engineering strategies to overcome.

Biological Barrier	Impact on Drug Delivery	Diseases Where Most Relevant	Nanomedicine‐Based Solutions	Reference
Aerodynamic deposition constraints	Limits delivery to distal lung regions; increases upper‐airway deposition	All pulmonary diseases	Nanoembedded microparticles, porous particles, optimized aerosol engineering	[23, 24, 25]
Mucus barrier	Restricts particle diffusion and promotes mucociliary clearance	CF, COPD, chronic bronchitis, bronchiectasis, severe asthma	PEGylated nanoparticles, mucus‐penetrating formulations	[26, 27]
Pulmonary surfactant interactions	Alters nanoparticle dispersion, aggregation, and cellular uptake	Distal lung delivery, mRNA therapeutics	Surfactant‐compatible lipid formulations, optimized LNP design	[28, 29]
Respiratory epithelial barrier	Limits uptake of biologics and nucleic acid therapeutics	CF, COPD, fibrosis, gene therapy applications	Cell‐penetrating carriers, endosomal escape technologies	[30, 31]
Cellular heterogeneity	Different target cells require distinct delivery strategies	Fibrosis, emphysema, cancer	Targeted nanoparticles, ligand‐mediated uptake	[32, 33]
Alveolar macrophage clearance	Rapid removal of inhaled particles before target‐cell uptake	Most pulmonary diseases	Biomimetic carriers, stealth coatings, macrophage‐targeting strategies	[29, 34]
Inflammatory microenvironment	ROS, proteases, and NETs may destabilize therapeutics	COPD, ARDS, infections	ROS‐responsive and enzyme‐responsive nanomedicines	[35, 36]
Fibrotic extracellular matrix	Restricts diffusion into diseased tissue	IPF, fibrotic ILD	Penetration‐enhanced nanoparticles, fibroblast‐targeted delivery	[37, 38, 39, 40]
Tumor microenvironment	Elevated interstitial pressure and hypoxia limit penetration	Lung cancer	Tumor‐targeted and stimuli‐responsive nanocarriers	[41, 42]
Biofilms and intracellular pathogen reservoirs	Reduce antimicrobial penetration and efficacy	Bronchiectasis, NTM disease, tuberculosis	Liposomes, macrophage‐targeted systems, biofilm‐penetrating carriers	[43, 44, 45, 46]

Abbreviations: ALIS, amikacin liposome inhalation suspension; ASO, antisense oligonucleotide; CF, cystic fibrosis; CFTR, cystic fibrosis transmembrane conductance regulator; LNP, lipid nanoparticle; MAC, Mycobacterium avium complex; mRNA, messenger RNA; RSV, respiratory syncytial virus; siRNA, small interfering RNA.

**FIGURE 1:**
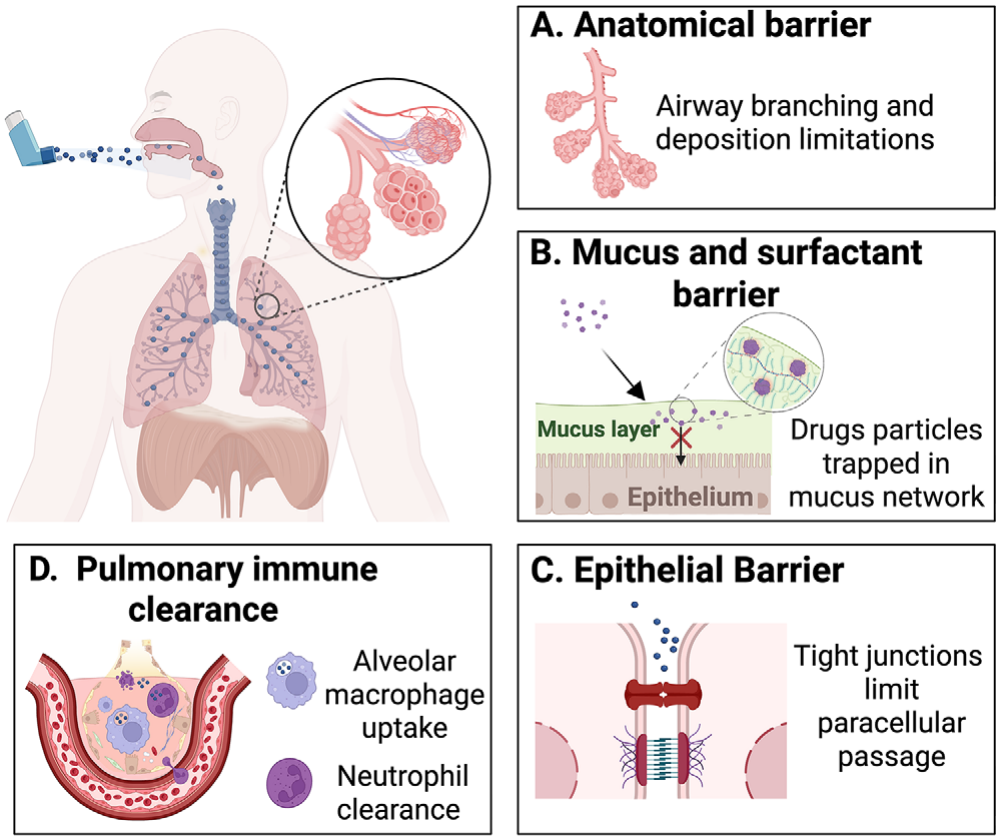
Biological barriers limiting pulmonary drug delivery. The schematic highlights major barriers that restrict effective inhaled drug delivery, including airway branching and deposition limitations, mucus/surfactant entrapment, epithelial tight junctions, and pulmonary immune clearance by macrophages and neutrophils. Together, these barriers reduce drug penetration and limit the amount of therapeutic agent reaching the intended lung target. Figure created using *Biorender.com*.

### 2.1 Aerosol Deposition and Anatomical Constraints

The respiratory tract is a highly branched structure in which successful inhalation depends on the aerodynamic behavior of the administered aerosol rather than on nanocarrier diameter alone [21, 22]. The primary therapeutic carrier may be nanoscale, but the material entering the airways can instead be a nebulized droplet containing nanoparticles, an aggregate, a spray‐dried nano‐in‐microparticle, or a porous microparticle. These two dimensions should therefore be considered separately. Physical diameter describes the geometric size of the therapeutic carrier, whereas aerodynamic diameter reflects the settling behavior of the inhaled particle or droplet and therefore determines how it behaves within the respiratory airstream [23]. Thus, a nanoscale carrier does not necessarily behave as a nanoscale aerosol during inhalation.

An aerodynamic diameter of approximately 1–5 µm is commonly targeted for lower‐airway and pulmonary deposition, although the actual deposition pattern depends on particle density, morphology, hygroscopic growth, inspiratory flow, airway geometry, and device performance [24].

Aerosol distributions are commonly characterized by the mass median aerodynamic diameter (MMAD) and geometric standard deviation (GSD), while the fine‐particle fraction (FPF) provides an estimate of the aerosol fraction within a respirable aerodynamic range [23]. Disease can substantially alter these relationships. Airway narrowing, mucus obstruction, and heterogeneous ventilation in obstructive lung disease produce uneven deposition and reduce access to distal targets [8, 25, 47, 48]. Once deposition has occurred, the nanoscale properties of the released carrier become more important than its preceding aerosol aerodynamic behavior. Nanocarrier size and surface characteristics influence diffusion through mucus, interactions with pulmonary surfactant, macrophage recognition, cellular uptake, intracellular trafficking, and penetration into diseased tissue. Nano‐in‐microparticle and porous‐particle formulations therefore provide a useful engineering bridge between efficient aerosol deposition and nanoscale biological delivery [23, 30, 49, 50, 51, 52, 53].

### 2.2 Mucus and Surfactant Barriers

Even when particles reach the desired region of the lung, they must first navigate the airway surface layers [8]. Mucus serves as an effective defense mechanism by trapping inhaled pathogens and particulates before their removal through mucociliary clearance (Figure 1) [54]. For drug delivery, however, this protective barrier can substantially limit particle mobility and residence time [55].

The challenge becomes particularly evident in diseases characterized by mucus hypersecretion, including cystic fibrosis, bronchiectasis, chronic bronchitis, and severe asthma [26, 56]. Increased mucin concentrations, extracellular DNA released from inflammatory cells, and cellular debris transform mucus into a dense viscoelastic network that restricts nanoparticle diffusion [57]. Early pulmonary gene delivery studies highlighted this problem when otherwise promising vectors exhibited poor access to airway epithelial cells despite adequate lung deposition [58]. Subsequent work demonstrated that densely PEGylated nanoparticles diffused more efficiently through cystic fibrosis sputum than conventional mucoadhesive systems, establishing a design principle that continues to influence pulmonary nucleic acid delivery [27]. Pulmonary surfactant presents a different challenge within the alveolar compartment [59]. Interest in surfactant‐nanoparticle interactions increased with the emergence of inhaled lipid nanoparticle platforms, where investigators observed notable differences between in vitro transfection efficiency and pulmonary performance in vivo [60, 61]. Surfactant phospholipids and proteins can influence particle dispersion, aggregation, protein corona formation, and cellular uptake [28]. As inhaled messenger RNA (mRNA) and gene‐editing therapies continue to advance, understanding these interactions has become increasingly important for optimizing delivery to distal lung regions.

### 2.3 Epithelial Barriers

Following transit through mucus and surfactants, therapeutics must cross the respiratory epithelium. While the alveolar barrier is remarkably thin, it remains highly selective and restricts the transport of many biologics and nanoparticle‐based therapeutics (Figure 1) [34, 62].

This limitation has become particularly apparent with inhaled RNA therapies. Formulations capable of producing robust gene expression following systemic administration frequently demonstrate reduced activity when delivered through the pulmonary route [61]. In many cases, the obstacle is not deposition but insufficient epithelial uptake, inefficient endosomal escape, or intracellular degradation of therapeutic cargo [31]. Consequently, improving intracellular trafficking has become as important as improving aerosol performance.

Cellular heterogeneity adds another layer of complexity. The respiratory epithelium contains multiple specialized cell populations with distinct uptake characteristics. For example, airway epithelial cells represent primary targets in cystic fibrosis, whereas alveolar type II (AT2) cells have emerged as important targets in regenerative approaches for emphysema and pulmonary fibrosis [32, 33, 58]. These differences help explain why delivery systems that perform well in one disease setting may be less effective in another.

### 2.4 Pulmonary Immune Clearance

The lung possesses highly efficient innate immune defenses that rapidly recognize and eliminate foreign material. Although essential for host protection, these mechanisms can substantially reduce the effectiveness of inhaled therapeutics.

Alveolar macrophages represent one of the most important barriers to pulmonary nanomedicine. Positioned along the alveolar surface, these cells continuously clear inhaled particles through phagocytosis [34]. Several studies evaluating inhaled lipid nanoparticles and polymeric carriers have reported substantial nanoparticle accumulation within macrophages shortly after administration, often limiting delivery to epithelial cells and other intended targets [29, 61]. This phenomenon has emerged as a recurring challenge in pulmonary nucleic acid delivery, where successful deposition does not necessarily translate into productive transgene expression.

Neutrophilic inflammation introduces additional complications. Conditions such as COPD, cystic fibrosis, ARDS, and severe bacterial pneumonia are characterized by elevated levels of reactive oxygen species, proteases, and neutrophil extracellular traps (NETs) [35, 36]. These factors can influence nanoparticle stability, alter drug release profiles, and physically restrict tissue penetration. Recognition of this challenge has stimulated the development of inflammation‐responsive carriers that exploit oxidative or enzymatic signals within diseased tissue to trigger localized drug release [63, 64].

### 2.5 Disease‐Specific Barriers

The barriers described above rarely occur in isolation. Instead, they are often amplified by disease‐specific pathological changes that can dramatically alter therapeutic performance.

Pulmonary fibrosis presents one of the most challenging environments for inhaled drug delivery. Excessive extracellular matrix deposition, tissue stiffening, and destruction of normal alveolar architecture increase diffusion distances and limit particle penetration into fibrotic regions [37]. Several inhaled antifibrotic nano formulations have demonstrated prolonged pulmonary retention in preclinical models, yet effective penetration into dense fibrotic tissue remains a significant obstacle [38].

Lung tumors create a different set of barriers. Elevated interstitial fluid pressure, hypoxia, abnormal vasculature, and immunosuppressive stromal populations collectively restrict intratumoral drug distribution [42]. This limitation has motivated the development of inhaled chemotherapeutics, RNA therapeutics, and immunomodulatory nanomedicines designed to improve local drug exposure while minimizing systemic toxicity.

Chronic inflammatory diseases such as asthma and COPD introduce additional variability through mucus hypersecretion, epithelial remodeling, oxidative stress, and immune‐cell infiltration [35]. ALI and ARDS can further disrupt regional ventilation through edema, alveolar flooding, and ventilation heterogeneity, thereby complicating aerosol delivery in critically ill and mechanically ventilated lungs [65].

In chronic pulmonary infections, biofilm formation presents a major obstacle to antimicrobial therapy. Biofilm‐associated pathogens such as *Pseudomonas aeruginosa* are embedded within extracellular matrices that restrict antibiotic penetration and contribute to treatment resistance [43]. The clinical success of liposomal amikacin for inhalation illustrates how nanotechnology can improve local drug retention and delivery within infected airways [44, 45]. As a whole, these examples highlight the need to design inhaled nanomedicines not only for the lung as an organ, but also for the unique microenvironment created by individual disease states.

The dominant delivery barrier varies across pulmonary diseases. Fibrosis is characterized by matrix accumulation, tissue stiffening, and distorted alveolar architecture that restrict tissue penetration. COPD is dominated by mucus hypersecretion, impaired mucociliary clearance, airway narrowing, and heterogeneous ventilation, affecting both aerosol deposition and post‐deposition transport. In ARDS, alveolar flooding, protein‐rich edema, barrier disruption, and severe ventilation heterogeneity create a distinct delivery environment. Formulation development should therefore reproduce the disease‐specific barriers most relevant to the intended pulmonary target. Beyond these structural constraints, pulmonary diseases may also involve altered chemoreflex control, adding further physiological heterogeneity during injury and recovery [66].

### 2.6 Disease‐Relevant Models for Formulation Screening

Conventional monocultures do not adequately reproduce the complex microenvironments encountered by inhaled nanomedicines. Disease‐relevant models can therefore improve formulation screening before in vivo evaluation. Patient‐derived airway organoids reproduce differentiated epithelial features, including mucus production and ciliary function, and COPD organoids can capture disease‐associated epithelial abnormalities [67]. Fibrotic alveolar organoids similarly provide a platform for evaluating therapeutic responses within a disease‐relevant alveolar environment [68].

Multicellular co‐cultures incorporating epithelial, endothelial, and macrophage populations can assess nanoparticle uptake, barrier transport, and inflammatory responses [69]. Lung‐on‐chip systems further reproduce epithelial–endothelial interfaces and breathing‐associated mechanical forces under dynamic conditions [70]. Together, these models can complement animal studies by enabling early evaluation of nanocarriers against disease‐specific mucus, epithelial, vascular, inflammatory, and mechanical barriers. Animal studies should also consider sex as a biological variable, because experimental ALI models have demonstrated sex‐dependent differences in ventilatory recovery despite chemoreflex sensitization in both sexes [71].

## 3 Nanomedicine Platforms for Inhaled Drug Delivery

Advances in nanotechnology have expanded the range of therapeutics that can be delivered through the pulmonary route (Figure 2). Modern inhaled nanomedicines are designed not only to improve drug solubility and retention but also to overcome biological barriers, enhance cellular uptake, and enable targeted delivery to diseased tissues. Several nanocarrier platforms have emerged, each offering distinct advantages and limitations depending on the therapeutic cargo and disease indication (Table 2; Table S3).

**FIGURE 2:**
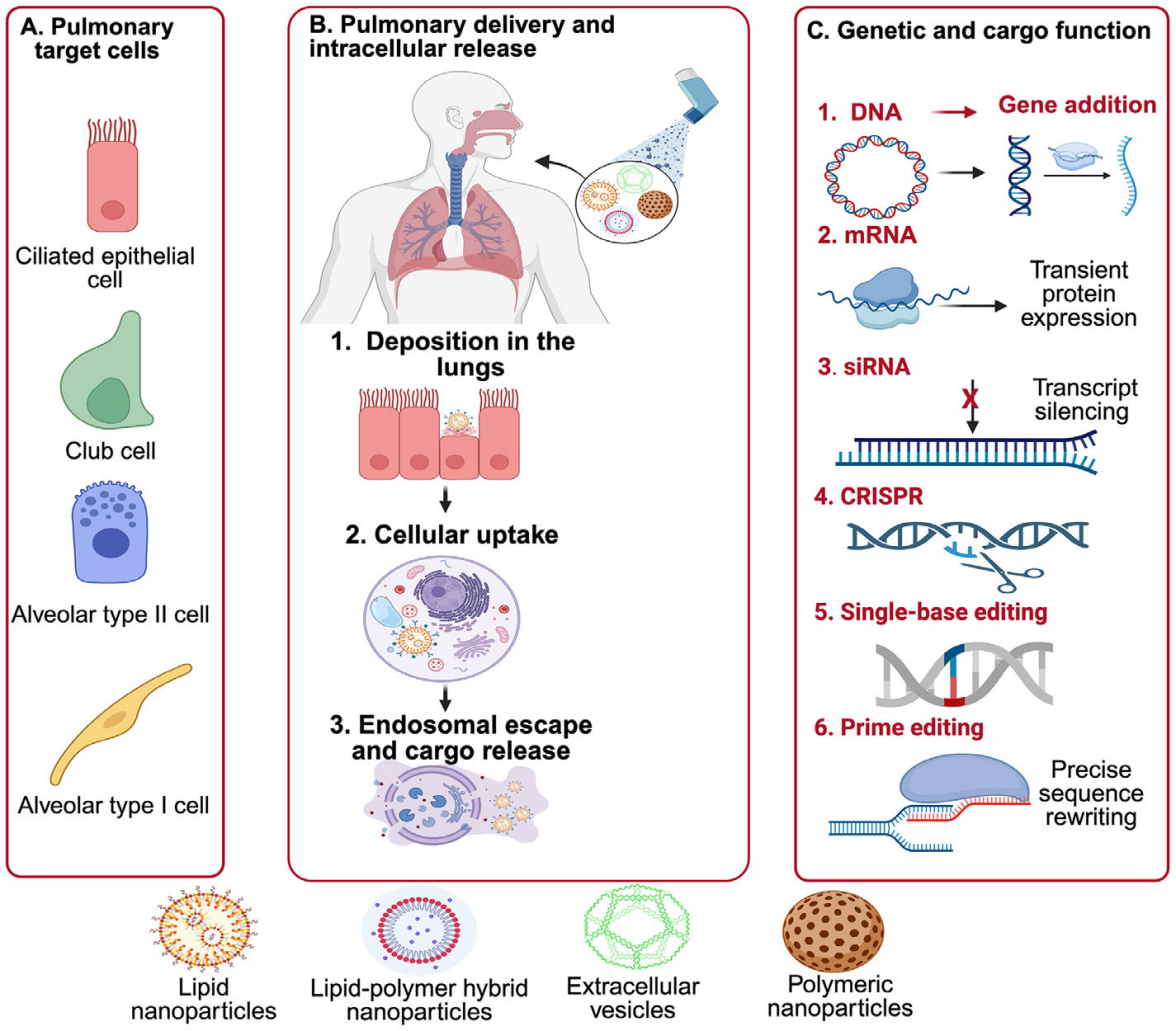
Future landscape of inhaled genetic tools. This schematic illustrates the inhaled delivery of genetic tools using nano/microcarrier platforms to target pulmonary cells. After aerosol deposition, cellular uptake, endosomal escape, and cargo release, different genetic cargos, including DNA, mRNA, siRNA, CRISPR, base editors, and prime editors, can enable gene addition, transient protein expression, transcript silencing, targeted gene editing, single‐base correction, or precise sequence rewriting for future lung therapies. Figure created using *Biorender.com*.

**TABLE 2:** Inhaled nanomedicine platforms: Key features, pulmonary applications, and translational status.

Platform	Representative Materials	Therapeutic Cargo	Major Advantages	Key Limitations	Most Relevant Pulmonary Applications	Translational Status
Liposomes	Phospholipids, cholesterol	Small molecules, antibiotics, proteins	Excellent biocompatibility; sustained drug release; established regulatory precedent	Drug leakage, limited nucleic acid loading, storage stability concerns	Pulmonary infections, antifibrotics, anti‐inflammatory therapies	Approved/Clinical [44, 45]
Lipid Nanoparticles (LNPs)	Ionizable lipids, cholesterol, PEG‐lipids	mRNA, siRNA, CRISPR cargo, small molecules	Highly efficient intracellular delivery; clinically validated for nucleic acids	Potential immunogenicity; formulation complexity; aerosolization challenges	Gene therapy, regenerative medicine, cancer immunotherapy, COPD/emphysema	Early clinical‐Clinical [72, 73, 74, 75]
Polymeric Nanoparticles	PLGA, chitosan, alginate, PEG‐based polymers	Small molecules, proteins, nucleic acids	Controlled release; tunable degradation; enhanced stability	Manufacturing complexity; slower regulatory progress	Antifibrotics, anti‐inflammatory therapies, sustained‐release formulations	Preclinical‐Early clinical [49, 50]
Hybrid Nanocarriers	Lipid–polymer composites	Small molecules, RNA therapeutics, biologics	Combines stability of polymers with cellular uptake of lipids	Scale‐up and manufacturing challenges	Lung cancer, fibrosis, nucleic acid delivery	Preclinical [76, 77]
Cell Membrane‐Coated Nanoparticles	Macrophage, leukocyte, erythrocyte, tumor‐cell membranes	Small molecules, proteins, nucleic acids	Immune evasion; prolonged retention; enhanced targeting	Complex manufacturing; batch variability	Inflammatory lung disease, fibrosis, cancer	Preclinical [78, 79]
Extracellular Vesicle (EV)‐Based Systems	Stem cell‐derived EVs, exosome‐inspired vesicles	RNA, proteins, regenerative factors	Natural biocompatibility; intrinsic cellular communication properties	Difficult purification; low production yield; cargo‐loading limitations	ALI/ARDS, fibrosis, regenerative therapies	Preclinical [80, 81]
Stimuli‐Responsive Nanomedicines	ROS‐, pH‐, enzyme‐, or hypoxia‐responsive materials	Small molecules, nucleic acids	Site‐specific drug release; reduced off‐target exposure	Increased formulation complexity; limited clinical experience	COPD, lung cancer, pulmonary infections, fibrosis	Preclinical [63, 82, 83]

### 3.1 Lipid‐Based Systems

Lipid‐based nanocarriers remain among the most clinically advanced platforms for pulmonary drug delivery due to their biocompatibility, scalability, and ability to encapsulate a wide range of therapeutic agents [84].

Liposomes were the first nanocarriers successfully translated into inhaled therapies. Their phospholipid composition resembles pulmonary surfactant, facilitating compatibility with the lung environment [59]. Liposomes can improve drug retention within the respiratory tract, reduce systemic exposure, and provide sustained release. The clinical approval of liposomal amikacin inhalation suspension for refractory nontuberculous mycobacterial lung disease represents a landmark achievement for inhaled nanomedicine and demonstrates the translational potential of lipid‐based carriers [44, 45]. Liposomal formulations have also been investigated for antifungal agents, antibiotics, corticosteroids, and anticancer drugs.

More recently, lipid nanoparticles (LNPs) have emerged as leading platforms for nucleic acid delivery. The success of mRNA vaccines accelerated interest in adapting LNP technology for pulmonary administration. However, inhaled delivery presents unique challenges not encountered with systemic administration, including mucus penetration, surfactant interactions, and macrophage uptake. Pulmonary performance is strongly influenced by lipid composition and surface chemistry. Ionizable and helper lipids, together with surface modifiers, affect colloidal stability, mucus and surfactant interactions, cellular tropism, and intracellular release. Nebulization can disrupt particle structure or reduce cargo retention, whereas optimized lipid architectures can preserve stability and pulmonary transfection [72, 73, 74]. Surface composition also influences target‐cell access, with engineered LNPs enabling airway epithelial and AT2‐directed delivery [75, 85]. Pulmonary LNP design should therefore optimize aerosol stability and target‐cell uptake together rather than focusing on encapsulation efficiency alone.

### 3.2 Polymeric Nanoparticles

Polymeric nanoparticles offer greater structural flexibility than many lipid‐based systems and can be engineered to achieve controlled drug release, improved stability, and enhanced tissue retention. Materials such as PLGA, chitosan, alginate, and PEG‐based copolymers have been extensively investigated for pulmonary delivery [49, 53].

One advantage of polymeric systems is the ability to precisely tailor particle properties through modifications in polymer composition, molecular weight, and surface chemistry. This versatility has enabled the development of carriers for small molecules, proteins, and nucleic acids. Nevertheless, concerns regarding biodegradation kinetics, manufacturing complexity, and potential pulmonary toxicity have limited clinical translation relative to lipid‐based platforms [86, 87]. As a result, polymeric nanoparticles continue to play a prominent role in preclinical development, particularly for applications requiring prolonged drug release or targeted intracellular delivery. Surface chemistry also creates a retention‐penetration trade‐off. Mucoadhesive polymers may prolong airway residence but can become restricted within dense mucus, whereas hydrophilic or near‐neutral surfaces generally favor mucus transport. Polymer design should therefore reflect whether the therapeutic objective requires proximal airway retention or penetration toward deeper epithelial targets [19, 27, 50].

### 3.3 Hybrid Nanocarriers

Hybrid nanocarriers seek to combine the favorable properties of multiple delivery systems within a single platform. Most commonly, these formulations integrate lipid and polymeric components to achieve improved stability while maintaining efficient drug loading and cellular uptake [88, 89].

The rationale behind hybrid systems stems from the observation that no single carrier platform adequately addresses all delivery challenges. Polymeric cores can provide structural integrity and controlled release, whereas lipid shells may improve biocompatibility and interactions with cellular membranes. Such designs have demonstrated promise for pulmonary delivery of chemotherapeutics, antimicrobial agents, and nucleic acids [76]. Although most hybrid systems remain at the preclinical stage, they illustrate the broader trend toward multifunctional carriers capable of addressing multiple delivery barriers simultaneously.

Hybrid systems allow carrier functions to be partially separated. For example, the polymeric components can provide structural stability and controlled release, whereas lipid domains can promote cellular interaction and RNA delivery. This added complexity can also increase formulation heterogeneity and reproducibility challenges, making post‐aerosolization characterization essential for inhaled products [50, 77]

### 3.4 Biomimetic Nanoparticles

Increasingly, researchers are drawing inspiration from endogenous biological systems to improve pulmonary drug delivery. Biomimetic nanoparticles are designed to exploit naturally evolved mechanisms for immune evasion, cellular communication, and tissue targeting.

Cell membrane‐coated nanoparticles represent one of the most widely investigated biomimetic strategies. By cloaking synthetic nanoparticles with membranes derived from erythrocytes, leukocytes, macrophages, or cancer cells, these systems can inherit surface proteins that reduce immune recognition and prolong retention within biological environments [79, 90]. In pulmonary applications, macrophage membrane‐coated nanoparticles have shown particular promise for inflammatory lung diseases because they can interact with inflammatory mediators while simultaneously delivering therapeutic cargo [78].

Extracellular vesicle (EV)‐inspired systems have also gained significant attention. EVs naturally mediate intercellular communication and possess intrinsic biocompatibility, making them attractive vehicles for drug and nucleic acid delivery (Figure 2) [91]. Several studies have demonstrated successful pulmonary delivery of EV‐based therapeutics in models of ALI, pulmonary fibrosis, and inflammatory lung disease [92, 93].

These biological interfaces also introduce translational liabilities. Cell‐membrane‐coated nanoparticles can vary with membrane source and preparation, while membrane integrity, batch consistency, sterilization, storage, and scale‐up require careful control. EV systems add heterogeneity in vesicle composition, cargo loading, purification yield, and source‐dependent variability. For inhaled formulations, preservation of the biological surface after aerosolization is particularly important because disruption or detachment of membrane components could alter targeting and immune interactions [80, 81, 90].

### 3.5 Stimuli‐Responsive Nanomedicines

Many pulmonary diseases are characterized by distinct pathological microenvironments, including elevated reactive oxygen species (ROS), altered pH, increased protease activity, or hypoxia [82, 83]. Stimuli‐responsive nanomedicines are designed to exploit these disease‐associated signals to trigger localized drug release.

ROS‐responsive systems have been particularly attractive for inflammatory lung diseases, in which oxidative stress directly contributes to disease progression. Similarly, pH‐sensitive and enzyme‐responsive carriers have been developed to improve drug release within infected tissues and tumor microenvironments [63, 83]. Rather than releasing cargo immediately after deposition, these systems aim to enhance therapeutic specificity by responding to biological cues present at the site of disease. Although most remain in preclinical development, stimuli‐responsive formulations represent an important step toward precision pulmonary drug delivery. Taken together, these platforms illustrate that successful inhaled nanomedicine depends less on a universally superior carrier than on matching material properties, biological interactions, aerosol stability, and therapeutic cargo to the intended pulmonary target. Because the delivery requirements of nucleic acids depend strongly on their molecular form and intracellular mechanism of action, mRNA, siRNA, antisense oligonucleotides, and genome‐editing cargos are compared separately in Section 5.

## 4 Disease‐Modifying Inhaled Nanomedicines for Major Pulmonary Diseases

Disease‐modifying inhaled nanomedicines must address different pathogenic mechanisms across pulmonary diseases rather than relying on a common delivery strategy. The following sections examine how local and cell‐directed delivery is being applied to fibrosis, lung cancer, COPD/emphysema, pulmonary infections, and ALI/ARDS, while highlighting the distinct biological barriers and translational constraints associated with each disease.

### 4.1 Pulmonary Fibrosis and Interstitial Lung Disease

Pulmonary fibrosis remains one of the most challenging chronic lung diseases to treat, with current therapies slowing disease progression rather than reversing established tissue damage (Table 3; Figure 3). The two approved antifibrotic agents, pirfenidone and nintedanib, have significantly improved disease management by reducing the rate of lung function decline and delaying progression in patients with IPF [94, 95]. Nevertheless, many patients continue to experience worsening fibrosis despite treatment, and adverse effects, including gastrointestinal intolerance and liver toxicity, frequently limit long‐term adherence.

**TABLE 3:** Disease‐specific nanomedicine strategies for precision pulmonary therapy.

Disease	Major Pathogenic Drivers	Primary Cellular Targets	Nanomedicine Strategy	Representative Therapeutic Cargo	Potential Disease‐Modifying Outcome	Translational Stage
Idiopathic Pulmonary Fibrosis (IPF)	TGF‐β signaling, fibroblast activation, profibrotic macrophages, epithelial dysfunction	Fibroblasts, macrophages, AT2 cells	Targeted nanoparticles, inhaled RNA therapeutics, biomimetic carriers	Pirfenidone, nintedanib, TGF‐β inhibitors, siRNA, antisense oligonucleotides, mRNA	Reduced fibrosis, suppression of fibroblast activation, enhanced epithelial repair	Preclinical/Early clinical [39, 40]
Lung Cancer	Tumor growth, immune evasion, hypoxia, abnormal tumor microenvironment	Tumor cells, tumor‐associated macrophages, T cells	Tumor‐targeted nanoparticles, inhaled immunotherapy, RNA delivery systems	Chemotherapy, immune stimulators, siRNA, mRNA, CRISPR cargo	Improved intratumoral drug delivery, enhanced antitumor immunity, reduced systemic toxicity	Preclinical/Clinical [41, 81]
COPD / Emphysema	Chronic inflammation, oxidative stress, cellular senescence, and impaired regeneration	AT2 cells, epithelial cells, macrophages, senescent cells	Antioxidant nanoparticles, senolytic delivery systems, regenerative gene therapy	Antioxidants, senolytics, FOXO3 modulators, TERT‐based therapies, mRNA, siRNA	Alveolar regeneration, restoration of repair pathways, attenuation of disease progression	Preclinical [33, 82, 96, 97]
Pulmonary Infections	Biofilms, intracellular pathogens, antimicrobial resistance, chronic inflammation	Airway epithelium, macrophages, infected cells	Liposomal antibiotics, biofilm‐penetrating nanocarriers, macrophage‐targeted delivery	Amikacin, ciprofloxacin, rifampicin, antimicrobial peptides, RNA therapeutics	Improved pathogen eradication, enhanced intracellular drug delivery, reduced resistance development	Clinically approved [44, 45, 98]
Acute Lung Injury (ALI) / ARDS	Cytokine storm, endothelial injury, epithelial barrier disruption, oxidative stress	Alveolar macrophages, endothelial cells, epithelial cells	Anti‐inflammatory nanoparticles, EV‐based therapies, RNA delivery platforms	Corticosteroids, cytokine inhibitors, siRNA, mRNA, regenerative biologics	Reduced inflammation, restoration of barrier integrity, accelerated tissue repair	Preclinical [80, 99]
Severe Asthma	Type 2 inflammation, epithelial injury, airway remodeling	Airway epithelial cells, eosinophils, immune cells	Targeted anti‐inflammatory nanoparticles, RNA therapeutics	Corticosteroids, anti‐cytokine therapies, siRNA	Improved local immune modulation and reduced airway remodeling	Preclinical [85]

**FIGURE 3:**
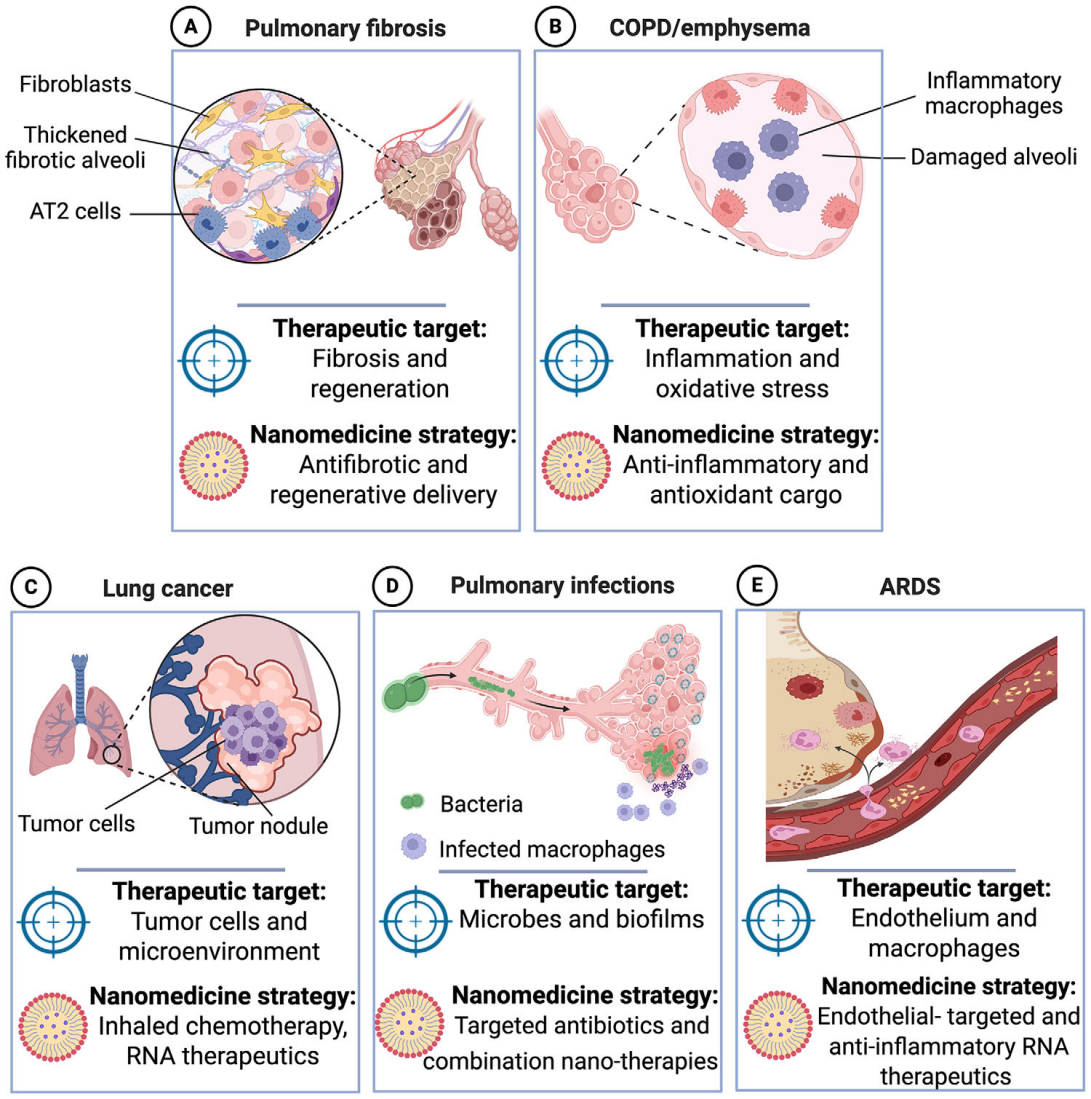
Disease‐specific applications of inhaled nanomedicine in pulmonary disorders. The figure highlights major lung diseases targeted by inhaled nanomedicine, including pulmonary fibrosis, COPD/emphysema, lung cancer, pulmonary infections, and ARDS. Each panel shows distinct pathological features that influence drug deposition, nanocarrier design, and site‐specific therapeutic delivery. Figure created using *Biorender.com*.

A major reason for this therapeutic gap is that fibrosis is sustained by multiple interacting cell populations rather than a single pathogenic pathway. Activated fibroblasts, dysfunctional alveolar epithelial cells, profibrotic macrophages, and excessive extracellular matrix deposition collectively drive disease progression [100]. As a result, systemically administered therapies often struggle to achieve sufficient concentrations within fibrotic tissue while simultaneously exposing healthy organs to off‐target effects. These limitations have stimulated growing interest in inhaled nanomedicines as a means of improving local drug delivery while reducing systemic toxicity.

Among emerging strategies, fibroblast‐directed therapies have received considerable attention because fibroblasts and myofibroblasts are the primary producers of pathological extracellular matrix. Several inhaled nanoparticle systems have been developed to improve pulmonary retention of antifibrotic agents or selectively deliver therapeutic cargo to fibroblast‐rich regions of the lung. In preclinical fibrosis models, inhaled nanocarrier formulations have improved pulmonary drug retention and attenuated fibrotic remodeling, supporting the therapeutic value of local carrier‐mediated delivery [101].

Transforming growth factor‐β (TGF‐β) remains a central target in pulmonary fibrosis because of its broad role in fibroblast activation and extracellular matrix production [102]. However, systemic inhibition of TGF‐β has proven difficult because the pathway is also essential for normal tissue homeostasis. Nanomedicine‐based approaches offer the possibility of localized pulmonary delivery of TGF‐β inhibitors, siRNA, or antisense oligonucleotides, thereby limiting systemic exposure. Several experimental studies have reported attenuation of fibrosis following targeted suppression of TGF‐β signaling, supporting continued interest in this approach.

The contribution of immune cells to fibrotic remodeling has also become increasingly apparent. Profibrotic macrophage populations can amplify fibroblast activation through sustained cytokine and growth‐factor signaling, creating a self‐perpetuating cycle of tissue remodeling [103]. Consequently, macrophage‐directed nanomedicines are being explored not simply as anti‐inflammatory therapies but as tools to interrupt disease‐driving cellular interactions. Although most remain in preclinical development, these strategies highlight a shift toward targeting the fibrotic microenvironment rather than individual signaling pathways alone.

More recently, attention has turned to the alveolar epithelium, particularly AT2 cells. Growing evidence suggests that impaired epithelial repair contributes directly to disease progression, raising the possibility that fibrosis may be addressed not only by suppressing matrix deposition but also by restoring regenerative capacity [32]. Advances in pulmonary delivery of mRNA, siRNA, and other nucleic acid therapeutics have made AT2 cells increasingly attractive targets for next‐generation interventions. While these approaches remain at an early stage, they represent a departure from traditional antifibrotic strategies and move the field closer to regenerative therapy.

The appeal of inhaled nanomedicines in pulmonary fibrosis is straightforward: the disease is localized to the lung, yet current therapies are administered systemically. Delivering therapeutics directly to the site of disease offers the potential to achieve higher local concentrations while minimizing dose‐limiting toxicities [62]. At the same time, advances in aerosol engineering, lipid nanoparticles, biomimetic carriers, and nucleic acid delivery systems have expanded the range of therapeutic modalities that can be administered through the pulmonary route.

Significant challenges remain. Fibrotic lungs exhibit distorted architecture, altered ventilation patterns, and dense extracellular matrix networks that may limit particle penetration [37]. Nevertheless, the convergence of targeted delivery technologies with a deeper understanding of fibrotic biology is creating opportunities that were not feasible a decade ago. As the field shifts from slowing disease progression toward modifying its underlying drivers, inhaled nanomedicines are emerging as a promising component of future therapeutic strategies for pulmonary fibrosis and other fibrotic interstitial lung diseases.

### 4.2 Lung Cancer

Lung cancer remains the leading cause of cancer‐related mortality worldwide despite substantial advances in molecularly targeted therapies and immunotherapy. Current treatment strategies include chemotherapy, targeted agents directed against actionable mutations such as EGFR and ALK, and immune checkpoint inhibitors (Table 3) [6]. Although these approaches have improved outcomes for selected patient populations, therapeutic efficacy is frequently limited by systemic toxicity, acquired resistance, and inadequate drug exposure within tumor tissue [42]. These challenges have generated growing interest in inhaled drug delivery as a strategy to increase local drug concentrations while minimizing off‐target effects (Figure 3).

Unlike most solid tumors, lung tumors can be accessed through both the vascular and airway routes, creating unique opportunities for localized therapeutic delivery. Unlike intravenous delivery, which relies on vascular transport and tumor penetration, inhaled therapeutics can be deposited directly within the respiratory tract and potentially achieve higher local concentrations at tumor sites. This approach may be particularly advantageous for patients with early‐stage disease, multifocal pulmonary lesions, or residual microscopic disease following conventional treatment.

Among the earliest applications of inhaled nanomedicine in lung cancer was the pulmonary delivery of chemotherapeutic agents. Conventional cytotoxic drugs such as cisplatin, paclitaxel, and doxorubicin are highly effective against tumor cells but are often associated with dose‐limiting systemic toxicities. Encapsulation within nanoparticles can improve pulmonary retention, prolong local drug exposure, and reduce systemic distribution. Several preclinical studies have demonstrated enhanced antitumor activity following inhalation of nanoparticle‐formulated chemotherapeutics compared with free‐drug counterparts, supporting continued development of localized treatment strategies [41].

The rapid expansion of cancer immunotherapy has further broadened interest in inhaled nanomedicines. While immune checkpoint inhibitors have transformed the treatment landscape for non‐small cell lung cancer, only a subset of patients achieve durable responses. The immunosuppressive tumor microenvironment remains a major barrier to therapeutic success. Inhaled delivery of immunomodulatory agents offers the possibility of reshaping local immune responses within the lung while limiting systemic immune‐related adverse effects. Experimental approaches have included inhaled cytokines, immune stimulators, and nanoparticle‐based platforms designed to enhance antitumor immunity directly within pulmonary tumors [104].

Nucleic acid therapeutics represent another rapidly evolving area. Advances in nanoparticle‐mediated delivery have enabled pulmonary administration of siRNA, mRNA, and other RNA‐based therapies capable of targeting oncogenic pathways that are difficult to address using conventional small molecules. Several preclinical studies have demonstrated successful pulmonary delivery and gene silencing using inhaled or intratracheal siRNA approaches, while mRNA‐based approaches are being explored to enhance antitumor immune responses and support combination immunotherapy strategies. Although clinical translation remains limited, these technologies offer considerable flexibility and may expand the range of actionable targets in lung cancer [105, 106].

A recurring challenge across all treatment modalities is the complex tumor microenvironment. Elevated interstitial pressure, abnormal vasculature, hypoxia, and dense extracellular matrix networks can restrict therapeutic penetration even when adequate drug concentrations are achieved at the tumor surface. To address these limitations, investigators have developed tumor‐targeted nanocarriers that exploit receptor‐mediated uptake, tumor‐associated enzymes, or microenvironment‐specific triggers to enhance local delivery [42]. Some platforms combine targeting ligands with controlled‐release mechanisms, allowing therapeutic activity to be concentrated within tumor tissue while reducing exposure to healthy lung regions.

Despite encouraging progress, relatively few inhaled nanomedicines for lung cancer have advanced to late‐stage clinical development. Regulatory considerations, manufacturing challenges, and concerns regarding long‐term pulmonary safety remain important barriers to translation. In addition, therapeutic performance must be evaluated within the highly heterogeneous environment of human lung tumors, which often differs substantially from preclinical models. Nevertheless, the convergence of inhalation technology, nanomedicine, and precision oncology is creating new opportunities for localized treatment. As delivery systems become increasingly sophisticated, inhaled nanomedicines may complement existing systemic therapies and contribute to more effective, less toxic treatment strategies for lung cancer.

### 4.3 COPD and Emphysema

COPD remains a leading cause of morbidity and mortality worldwide. While COPD encompasses multiple pathological processes, emphysema is characterized by progressive destruction of alveolar structures, loss of gas exchange surface area, and irreversible decline in lung function. Current pharmacological therapies improve symptoms and reduce exacerbations, yet none restore damaged alveoli or halt disease progression [16]. This unmet need has driven growing interest in inhaled nanomedicines designed to address the underlying biological mechanisms of disease rather than its clinical manifestations alone (Figure 3). The current therapeutic landscape is dominated by bronchodilators, inhaled corticosteroids, phosphodiesterase‐4 inhibitors, and, more recently, biologics targeting selected inflammatory pathways (Table 4). These therapies improve airflow limitation and quality of life but exert limited effects on the structural deterioration that defines emphysema. This limitation reflects the multifactorial nature of COPD, where chronic inflammation, oxidative stress, cellular senescence, mitochondrial dysfunction, and impaired tissue repair collectively contribute to disease progression [35, 97].

**TABLE 4:** Representative clinical trials of inhaled nanomedicines and genetic medicines for pulmonary diseases.

Therapeutic candidate / product	Disease / indication	Cargo / active agent	Target / mechanism of action	Delivery platform	Clinical phase / status	NCT Number	Key Clinical Findings
Amikacin liposome inhalation suspension (ALIS; Arikayce)	Treatment‐refractory *Mycobacterium avium* complex (MAC) lung disease	Amikacin	Inhibition of bacterial protein synthesis; sustained intracellular/local amikacin exposure against MAC	Liposome	Phase III; FDA‐approved	NCT02344004	Improved sputum culture conversion in refractory MAC lung disease [45]
ALIS‐based regimen (ENCORE)	Newly diagnosed MAC lung infection	Amikacin	Inhibition of bacterial protein synthesis against MAC	Liposome	Phase III	NCT04677569	Completed evaluation in newly diagnosed MAC lung disease
Liposomal ciprofloxacin / ARD‐3150 (ORBIT‐3)	Non‐CF bronchiectasis with chronic *Pseudomonas aeruginosa* infection	Ciprofloxacin	Bacterial DNA‐gyrase/topoisomerase inhibition; sustained local ciprofloxacin exposure	Liposomal ciprofloxacin dispersion	Phase III completed	NCT01515007	Did not significantly prolong time to first exacerbation [107]
Liposomal ciprofloxacin / ARD‐3150 (ORBIT‐4)	Non‐CF bronchiectasis with chronic *P. aeruginosa* infection	Ciprofloxacin	Bacterial DNA‐gyrase/topoisomerase inhibition; sustained local ciprofloxacin exposure	Liposomal ciprofloxacin dispersion	Phase III completed	NCT02104245	Significantly prolonged time to first exacerbation [107]
pGM169/GL67A	Cystic fibrosis	CFTR plasmid DNA	CFTR gene replacement	Cationic liposome gene‐transfer complex	Phase II completed	NCT01621867	Demonstrated modest stabilization of lung function [108]
MRT5005	Cystic fibrosis	CFTR mRNA	CFTR protein restoration through mRNA replacement	Lipid nanoparticle	Phase I/II completed	NCT03375047	Repeated nebulized administration was feasible, but no beneficial effect on FEV1 was demonstrated.
Eluforsen / QR‐010	Cystic fibrosis with F508del‐CFTR	Antisense oligonucleotide	Antisense‐mediated correction of CFTR transcript function	Inhaled RNA therapeutic	Phase Ib completed	NCT02532764	Safe and well tolerated in cystic fibrosis.
SPL84	Healthy volunteers; intended for Cystic fibrosis with 3849+10 kb C→T mutation	Antisense oligonucleotide	Antisense‐mediated CFTR splicing correction	Inhaled RNA therapeutic	Phase I completed	NCT06217952	Demonstrated favorable safety and pharmacokinetic profiles.
ALN‐RSV01	RSV infection in lung transplant recipients	siRNA targeting RSV nucleocapsid gene	RSV nucleocapsid gene silencing	Inhaled / intranasal RNAi therapeutic	Phase IIb completed	NCT01065935	Showed a trend toward reduced bronchiolitis obliterans syndrome progression after RSV infection [109]
siR‐7‐EM/KK‐46 / MIR 19	COVID‐19	SARS‐CoV‐2 siRNA	SARS‐CoV‐2 RNA‐dependent RNA polymerase inhibition through siRNA‐mediated viral RNA silencing	Peptide dendrimer formulation	Phase II; Phase IIb/III reported	NCT05184127; NCT05783206	Demonstrated acceptable safety; Clinical efficacy findings were dose‐dependent and mixed [110]

*Data source*: ClinialTrials.gov.

Among the earliest nanomedicine approaches investigated in COPD were anti‐inflammatory delivery systems. Encapsulation of corticosteroids and other anti‐inflammatory agents within nanoparticles can enhance pulmonary retention, reduce systemic exposure, and improve drug distribution within diseased tissue. More recently, inhaled siRNA and other nucleic acid‐based approaches have been explored to suppress inflammatory mediators that contribute to persistent lung injury [111]. While most remain in preclinical development, these studies demonstrate how nanocarriers can be used to improve the therapeutic index of existing pharmacological strategies.

Oxidative stress is another hallmark of COPD pathogenesis and represents an attractive target for disease modification. Excessive production of reactive oxygen species contributes to epithelial injury, extracellular matrix degradation, and accelerated cellular aging. Several inhalable nanoparticle formulations have been developed to improve pulmonary delivery of antioxidants or activate endogenous protective pathways, such as Nrf2 signaling [82, 112]. Rather than simply suppressing downstream inflammation, these approaches aim to intervene earlier in the pathological cascade.

The recognition of cellular senescence as a driver of emphysema has opened another promising therapeutic avenue. Senescent epithelial, endothelial, and immune cells accumulate within diseased lungs and secrete a range of proinflammatory mediators that reinforce tissue damage and impair repair mechanisms. Consequently, senolytic therapies capable of selectively eliminating senescent cells have attracted increasing attention [96]. Nanocarrier‐based delivery may be particularly advantageous in this setting by concentrating senolytic agents within the lung while limiting systemic toxicity, a challenge that has hindered the broader application of several senolytic compounds.

Perhaps the most transformative strategies are those aimed at restoring alveolar regeneration. Increasing evidence implicates AT2 cells as central regulators of alveolar maintenance and repair. Dysfunction of these progenitor‐like cells is increasingly recognized as a contributor to emphysema progression, making them attractive targets for next‐generation therapies. Advances in pulmonary nucleic acid delivery have enabled exploration of mRNA, siRNA, and gene‐modulating approaches designed to enhance epithelial survival and regenerative capacity. Pathways involved in cellular survival, progenitor‐cell maintenance, and alveolar regeneration, including FOXO3, telomerase reverse transcriptase (TERT), and Wnt/β‐catenin signaling, have emerged as particularly promising candidates for therapeutic modulation [33, 113]. Although most regenerative and gene‐based approaches remain at an early stage of development, they represent a fundamental departure from conventional COPD treatment. Rather than slowing functional decline, these strategies aim to restore the biological processes required for alveolar repair. The combination of inhaled delivery and nanomedicine is especially attractive because it allows therapeutic cargo to be concentrated within the diseased organ while minimizing systemic exposure.

Important challenges remain, including disease heterogeneity, mucus obstruction, and efficient delivery to distal alveolar regions. Nevertheless, advances in aerosol engineering, lipid nanoparticles, biomimetic carriers, and nucleic acid therapeutics are steadily expanding what can be achieved through the pulmonary route. As the field moves beyond symptom management toward regeneration and disease modification, inhaled nanomedicines may offer one of the most promising opportunities to fundamentally alter the treatment landscape for COPD and emphysema.

### 4.4 Pulmonary Infections, Biofilms, and Antimicrobial Resistance

Pulmonary infections remain difficult to treat because antimicrobial success depends on both drug potency and drug access to infected lung compartments (Figure 3). Even highly active antimicrobials may fail when pathogens reside within mucus‐obstructed airways, intracellular macrophage niches, necrotic lesions, or biofilm‐rich microenvironments that limit drug penetration. This challenge is particularly relevant in cystic fibrosis, bronchitis, tuberculosis, nontuberculosis mycobacterial disease, ventilator‐associated pneumonia, and chronic fungal infection. Antimicrobial resistance further narrows treatment options because repeated subtherapeutic exposure can select resistant organisms and allow a persistent microbial population to survive [5, 14].

Biofilms are a major reason for treatment failure in chronic lung infection. Bacteria within biofilms are protected by an extracellular matrix that slows antibiotic penetration and reduces immune clearance. Diseased lungs also show uneven ventilation, mucus plugging, impaired perfusion, and an altered epithelial barrier. These factors create regions where drug levels remain below effective concentration. The appeal of inhaled nanomedicine lies in its ability to increase local drug exposure while minimizing systemic toxicity. Depending on carrier design, nanomedicines can prolong airway residence, improve transport through airway secretions, facilitate intracellular delivery, and sustain antimicrobial release within infected tissues [46].

The most compelling clinical example is amikacin liposome inhalation suspension (ALIS), which became the first inhaled nanomedicine approved for the treatment of refractory *Mycobacterium avium* complex lung disease. In the phase 3 CONVERT trial, adding ALIS to guideline‐based therapy increased the proportion of patients achieving sputum culture conversion within 6 months compared with standard therapy alone [45]. Follow‐up studies demonstrated durable responses in a subset of patients, providing important clinical validation for localized nanomedicine‐based antimicrobial delivery [44].

Inhaled ciprofloxacin has also been evaluated in non‐cystic fibrosis bronchiectasis. Ciprofloxacin dry powder reduced bacterial burden in an early clinical study, while the RESPIRE trial demonstrated that clinical benefit may depend on dosing schedule, pathogen profile, and patient selection [114, 115, 116]. Clinical experience with liposomal ciprofloxacin further illustrates this translational variability. In the replicate phase III ORBIT‐3 and ORBIT‐4 trials, ARD‐3150 significantly prolonged the time to first pulmonary exacerbation in ORBIT‐4 but not in ORBIT‐3, and the pooled analysis did not reach statistical significance [107]. More recent analysis associated differences in treatment response with distinct *Pseudomonas aeruginosa* microbial and inflammatory endotypes, emphasizing disease heterogeneity as an important determinant of inhaled‐antibiotic efficacy [117].

Taken together, these findings show that pulmonary delivery by itself is not enough. In heterogeneous chronic infections, clinical benefit still depends on the context of disease, patient variability, and whether the delivered therapy addresses the main drivers of pathology.

Current research is increasingly focused on overcoming biological barriers that contribute to treatment failure, including biofilm penetration and intracellular delivery to infected macrophages. This is particularly relevant for tuberculosis and nontuberculous mycobacterial disease, where pathogens reside within cellular reservoirs that are often difficult to access using conventional therapy [118, 119]. Liposomes, polymeric nanoparticles, solid lipid carriers, and mesoporous systems can be designed to improve biofilm penetration, protect unstable antimicrobials, and enhance uptake by infected macrophages [49, 120, 121]. This is highly relevant for tuberculosis and intracellular bacterial infection, where macrophage‐targeted delivery may raise drug levels inside the microbial niche [98, 121]. Emerging strategies also combine antibiotics with anti‐biofilm agents, quorum‐sensing inhibitors, efflux‐pump inhibitors, or RNA‐based therapeutics. Considered together, these approaches reflect a shift from simply delivering higher antibiotic concentrations toward engineering multifunctional systems capable of addressing the biological mechanisms underlying chronic infection and antimicrobial resistance.

### 4.5 ALI and ARDS

Despite decades of research, pharmacological management of ALI and ARDS remains largely supportive. Mechanical ventilation, prone positioning, conservative fluid management, and treatment of the underlying trigger improve outcomes, yet no approved therapy directly restores the damaged alveolar‐capillary barrier or accelerates repair of injured lung tissue. This persistent therapeutic gap has renewed interest in inhaled nanomedicines capable of delivering anti‐inflammatory, barrier‐protective, and regenerative therapies directly to injured alveoli (Figure 3). Experimental studies further indicate that respiratory dysfunction may persist beyond the acute phase, with sustained chemoreflex sensitization and sex‐dependent differences in ventilatory recovery following ALI [122].

The pulmonary route is particularly attractive because injury is largely confined to the lung, creating an opportunity to achieve high local drug concentrations while limiting systemic toxicity.

Inhaled nanocarriers may improve delivery of therapeutics to damaged alveolar regions and inflamed pulmonary tissue. At the same time, pulmonary edema, alveolar flooding, and regional ventilation abnormalities can alter aerosol deposition, highlighting the need for delivery systems that remain effective under pathological conditions. This makes ALI/ARDS an important setting for inhaled nanomedicine, particularly for strategies designed to deliver anti‐inflammatory, barrier‐protective, or macrophage‐targeted therapies directly to injured lung regions [123].

Endothelial‐targeted carriers have attracted considerable attention because vascular leakage is a defining feature of ALI/ARDS. By stabilizing endothelial junctions and reducing permeability, these approaches aim to interrupt one of the earliest drivers of respiratory failure.

Inhaled nanocarriers are also of interest because local delivery may increase drug exposure at damaged alveolar sites while limiting systemic toxicity. Endothelial‐targeted carriers could help stabilize the vascular barrier and reduce leakage. A macrophage‐directed strategy is particularly relevant in ALI/ARDS because macrophages contribute to both inflammatory amplification and resolution [123]. Inhalable lipid nanomedicines have demonstrated preclinical benefit by modulating macrophage polarization and suppressing inflammatory signaling pathways, including TLR4/MyD88/NF‐κB signaling [99].

RNA‐based therapeutics represent another emerging strategy. By selectively regulating inflammatory mediators or enhancing protective cellular pathways, inhaled RNA delivery may offer a level of therapeutic precision that is difficult to achieve using conventional anti‐inflammatory drugs. Several experimental studies have reported encouraging results using inhaled nucleic acid platforms to suppress inflammatory transcripts or enhance tissue‐protective signaling [124]. Extracellular vesicle (EV)‐based inhalation therapies have generated growing interest because of their ability to modulate multiple pathological processes simultaneously. Preclinical studies suggest that EVs may attenuate inflammation, reduce cytokine storm responses, improve mitochondrial function, and promote tissue repair following ALI [80]. Unlike many single‐target therapies that have failed in ARDS, EV‐based approaches may exert broader effects across several interconnected pathways involved in lung injury and recovery.

Despite encouraging progress, translation into clinical practice remains challenging. Rapid disease progression, critically ill patient populations, ventilator‐compatible aerosol delivery systems, and stringent safety requirements create barriers that are less prominent in chronic lung diseases. Nevertheless, advances in nanocarrier engineering, RNA therapeutics, and EV technologies continue to strengthen the rationale for inhaled approaches capable of directly targeting the biological drivers of ALI and ARDS. These clinical experiences highlight why strong preclinical pulmonary exposure does not necessarily translate into human efficacy. Patient‐specific airway architecture and disease heterogeneity can alter regional deposition and target‐cell access, while formulation‐device interactions can change delivered dose and carrier integrity. Repeated administration may additionally reveal inflammatory or hypersensitivity responses that are poorly predicted by animal models [9, 23, 50, 125]. Clinical translation therefore requires simultaneous consideration of patient phenotype, device performance, local pharmacology, and repeat‐dose tolerability.

## 5 Inhaled Nucleic Acid Therapeutics: Matching Molecular Mechanism to Pulmonary Delivery

Nucleic acid therapeutics place different requirements on pulmonary delivery because their molecular forms, intracellular destinations, durations of action, and safety liabilities are not equivalent. Successful inhalation therefore requires more than depositing nucleic acid in the lung. The cargo must remain intact during aerosol generation, cross disease‐dependent mucus or surfactant barriers, enter the relevant cell population, escape non‐productive intracellular compartments, and reach either the cytoplasm or nucleus in an active form [126]. These requirements differ substantially among mRNA replacement, siRNA‐mediated silencing, antisense‐mediated splicing modulation, and genome editing. Accordingly, the appropriate carrier should be selected around both the intended lung cell and the molecular mechanism of the cargo rather than around nucleic acid delivery as a single platform [124, 126].

### 5.1 mRNA Replacement

mRNA replacement provides transient protein expression without permanent genomic modification. Its relatively large, single‐stranded structure requires protection from extracellular degradation and efficient cellular uptake, followed by endosomal escape because translation occurs in the cytoplasm [127, 128]. These requirements have made lipid nanoparticles (LNPs) attractive for pulmonary mRNA delivery, although formulations optimized for systemic administration do not necessarily retain their stability or transfection efficiency after aerosolization.

Cystic fibrosis provides the most clinically advanced example. Nebulized CFTR mRNA has been evaluated as a genotype‐independent strategy for restoring CFTR expression, but the phase 1/2 MRT5005 study did not establish a clear improvement in lung function despite demonstrating the feasibility of repeated inhaled mRNA administration [125]. This outcome is important because it separates successful pulmonary administration from successful therapeutic delivery: deposition in the lung does not ensure sufficient expression in the epithelial population required for functional correction [125].

Recent preclinical studies have therefore focused on preserving carrier integrity during nebulization while improving cellular uptake and intracellular delivery. Optimized LNPs, degradable ionizable lipids, inhalable mRNA nanoparticles, and dry‐powder formulations have improved pulmonary expression in experimental systems [29, 72, 73, 74, 129, 130]. The transient nature of mRNA offers reversibility, but chronic diseases are likely to require repeated administration. Clinical utility will therefore depend on balancing expression level and duration against dosing frequency, carrier‐associated inflammation, and airway tolerability [131].

### 5.2 siRNA Mediated Gene Silencing

siRNA differs from mRNA in both molecular scale and pharmacological objective. It is a short double‐stranded RNA that acts in the cytoplasm to reduce expression of a selected transcript rather than to supply a protein. Protection from extracellular degradation, cellular uptake, and endosomal escape remain essential, but therapeutic success is determined by the degree and duration of target knockdown rather than by protein production.

Early clinical studies with ALN‐RSV01 established proof of concept for respiratory RNA interference. The therapy showed antiviral activity in a controlled respiratory syncytial virus challenge study and was subsequently evaluated in lung‐transplant recipients with RSV infection [109, 132]. Pulmonary siRNA has since expanded into non‐infectious disease. Inhaled nanoparticles targeting IL‐11 reduced collagen deposition and improved lung function after bleomycin injury, while a newer formulation increased retention within fibrotic lesions and suppressed disease progression [39, 40]. Airway epithelial delivery has also been demonstrated with LNPs carrying siRNA against TSLP in experimental asthma [85]. These studies illustrate a key design constraint for siRNA which shows that a formulation can deposit efficiently in the lung but remain ineffective if it fails to reach the cell population producing the pathogenic transcript. For chronic indications, the reversible nature of gene silencing permits dose adjustment but also creates a requirement for sustained or repeated exposure.

### 5.3 Antisense‐Mediated Splicing Modulation

Antisense oligonucleotides (ASOs) provide a mechanistically distinct approach to pulmonary genetic therapy. These short, chemically modified single‐stranded oligonucleotides can alter RNA processing rather than supply a protein or promote transcript degradation. Splice‐modifying ASOs must ultimately gain access to nuclear pre‐mRNA, giving them a different intracellular destination from cytoplasmic mRNA translation and siRNA activity [124, 133].

Cystic fibrosis provides the clearest pulmonary application. Inhaled programs including eluforsen and SPL84 have progressed into clinical evaluation for defined CFTR variants (Table 4). Their development illustrates an important trade‐off. The relatively compact and chemically stabilized nature of ASOs can reduce some formulation demands associated with larger RNA cargos, but efficient epithelial uptake, intracellular trafficking, sequence‐dependent toxicity, and the need for repeated administration remain limiting factors. ASO therapy is therefore most compelling when a genetically defined patient population can be matched to a specific RNA‐processing defect.

### 5.4 Genome Editing

Genome editing differs fundamentally from mRNA, siRNA, and ASO therapy because transient exposure to the therapeutic machinery can produce a persistent change in genomic DNA. This potential durability may reduce the need for repeated treatment, but it also increases the consequences of editing unintended cells or genomic sites. Pulmonary genome editing therefore places particularly stringent demands on cell selectivity, intracellular delivery, and safety.

#### 5.4.1 CRISPR Nuclease Editing

CRISPR nuclease systems can be delivered in different molecular formats, including nucleic acid components or preassembled ribonucleoprotein complexes. Productive editing requires that the editing machinery reaches the nucleus of the relevant cell. A recent LNP platform delivered engineered Cas9 ribonucleoproteins and achieved editing of disease‐associated SFTPC in lung tissue, supporting the feasibility of nonviral lung‐directed genome editing after systemic LNP delivery [134]. Compared with reversible RNA therapeutics, however, CRISPR nuclease editing introduces additional concerns related to double‐strand DNA breaks, off‐target cleavage, and editing of unintended cell populations.

#### 5.4.2 Base Editing

Base editing permits selected nucleotide substitutions without requiring a conventional nuclease‐induced double‐strand break. Adenine base editors have corrected disease‐associated CFTR mutations in human airway epithelial cells. More recently, Dual SORT LNPs enabled systemic multi‐organ base editing, including editing within lung AT2 cells in a model relevant to alpha‐1 antitrypsin deficiency [75]. These results expand the range of pulmonary mutations that may be therapeutically accessible, but the delivery burden remains substantial because the editor and guide RNA must reach the same cell and gain nuclear access. Bystander editing and genomic off‐target activity also remain important safety considerations.

#### 5.4.3 Prime Editing

Prime editing can address sequence changes that are not readily corrected through conventional base editing, including more complex CFTR variants. Recent studies have restored CFTR‐related function in human airway epithelial cells and organoid models and have optimized prime‐editing systems for F508del correction [135, 136, 137]. This broader editing scope is accompanied by increased delivery complexity because both the prime editor and prime‐editing guide RNA must be delivered efficiently to the same target cell. Pulmonary prime editing therefore remains predominantly preclinical, with co‐delivery efficiency, nuclear access, aerosol‐compatible formulation, and editing specificity representing major translational barriers.

### 5.5 Target‐Cell Selectivity and Productive Delivery

Target‐cell identity is a central determinant of whether pulmonary nucleic acid delivery succeeds or fails. Airway epithelial cells are required for most CFTR‐directed strategies, whereas AT2 cells are relevant to surfactant disorders and selected regenerative or fibrotic applications. Macrophages may be desirable targets in inflammatory and infectious diseases but can function as a competing particle sink when epithelial transfection is required. Tumor‐directed RNA delivery faces a different problem, as heterogeneous lesions and regional airway access can produce highly variable exposure [124].

These distinctions explain why high total lung uptake does not necessarily predict therapeutic activity. A formulation that accumulates efficiently in alveolar macrophages may perform poorly when the desired target is airway or alveolar epithelium. Conversely, deposition in the correct anatomical region may still fail to generate activity because of inadequate cellular internalization or endosomal escape. Cell‐selective delivery should therefore be treated as a design variable linking disease biology to carrier chemistry rather than as a downstream biodistribution measurement. The airway epithelial cell‐specific siRNA delivery achieved with targeted LNPs illustrates how changing cellular tropism can directly alter therapeutic performance [85].

### 5.6 Aerosol Stability, Repeat Dosing, and Translational Trade‐offs

Before any nucleic acid modality reaches its intracellular site of action, the formulation must survive aerosol generation. Nebulization exposes carriers to shear forces and air‐liquid interfaces that can change particle size, promote aggregation or cargo leakage, and reduce biological activity [138, 139].

After deposition, mucus, surfactant, mucociliary clearance, macrophage uptake, and endosomal sequestration create additional barriers between regional lung delivery and productive intracellular exposure [124, 133]. The intracellular requirement also differs among modalities. mRNA and siRNA require efficient cytoplasmic release, whereas splice‐modifying ASOs and genome‐editing systems require access to nuclear targets. mRNA, siRNA, and ASOs generally produce reversible pharmacological effects, which permits dose adjustment but increases dependence on repeated administration. Repeated inhalation can increase cumulative concerns related to innate immune sensing, airway inflammation, and carrier‐associated toxicity [133, 139].

Genome editing may provide longer‐lasting activity after fewer administrations, but this advantage is offset by stricter requirements for target‐cell specificity and control of persistent off‐target consequences. Manufacturing requirements similarly increase with molecular complexity. Short synthetic oligonucleotides, long mRNA cargos, multicomponent LNP formulations, and genome‐editing complexes differ substantially in production, characterization, stability, and quality‐control requirements [124, 139]. Their clinical maturity is also uneven. Inhaled mRNA and siRNA have entered human studies, and splice‐modifying ASOs have reached clinical evaluation, whereas pulmonary CRISPR nuclease, base‐editing, and prime‐editing strategies remain predominantly preclinical. The relevant translational question is therefore not which nucleic acid modality is intrinsically superior, but whether its mechanism, target cell, duration of action, delivery burden, aerosol stability, and safety profile are compatible with the disease being treated.

Across pulmonary applications, these modalities differ principally in therapeutic duration and delivery burden. mRNA is suited to transient protein replacement, whereas siRNA provides reversible suppression of disease‐associated transcripts and generally requires repeated dosing to maintain knockdown. Genome editing offers the possibility of durable correction but places greater demands on target‐cell specificity and control of off‐target effects. The appropriate modality therefore depends on disease mechanism, target‐cell accessibility, required duration of action, repeat‐dose tolerability, and the consequences of unintended activity.

## 6 Clinical Translation and Regulatory Considerations

Despite remarkable advances in pulmonary nanomedicine over the past two decades, relatively few inhaled nanomedicines have reached clinical practice. While numerous platforms have demonstrated promising preclinical efficacy, successful translation requires overcoming challenges that extend beyond biological performance. Manufacturing scalability, aerosol delivery, long‐term safety, regulatory expectations, and clinical trial design all influence whether a promising laboratory technology ultimately becomes a therapeutic product.

### 6.1 Manufacturing Challenges

Manufacturing remains one of the most significant barriers to clinical translation. Nanomedicines often possess complex physicochemical characteristics that must be tightly controlled to ensure reproducibility, stability, and therapeutic performance. Parameters such as particle size distribution, surface charge, drug loading efficiency, encapsulation stability, and release kinetics can directly influence biological activity and regulatory acceptance [86, 140].

For GMP‐scale production, critical quality attributes should be linked to predefined acceptance criteria, including particle‐size distribution, polydispersity, drug or cargo content, encapsulation efficiency, free versus carrier‐associated cargo, physical stability, sterility, and batch‐to‐batch reproducibility. For inhaled products, these controls should be complemented by post‐aerosolization measurements of emitted dose and aerodynamic performance.

These challenges become particularly pronounced for inhaled formulations because nanoparticles must retain their properties during both manufacturing and aerosolization. Batch‐to‐batch variability that may be acceptable in early‐stage research can become problematic during large‐scale production. In addition, advanced platforms such as LNPs, biomimetic carriers, and EV‐based therapeutics frequently require specialized manufacturing processes that remain difficult to scale economically [141]. Recent progress in microfluidic manufacturing, continuous production technologies, and automated quality‐control systems has improved scalability for several nanoparticle platforms. Nevertheless, reproducible large‐scale production remains a critical hurdle for many next‐generation inhaled nanomedicines.

### 6.2 Aerosolization and Device Compatibility

Unlike systemically administered nanomedicines, an inhaled product must be developed as a formulation‐device combination. Nebulizers, dry powder inhalers, and pressurized metered‐dose inhalers generate aerosols through different mechanisms, and a carrier that is biologically effective before aerosolization may not retain the same properties after delivery [142, 143]. Formulation composition, aerodynamic behavior, and device selection must therefore be optimized together rather than evaluated as independent variables [23, 50].

Aerosol performance should be assessed using complementary measures that describe both aerosol quality and the amount of material available for inhalation. MMAD and GSD define the aerodynamic size distribution, whereas fine‐particle fraction and respirable fraction estimate the proportion capable of reaching therapeutically relevant regions. Emitted dose, delivered dose, aerosol output rate, and regional deposition efficiency provide additional information on how much of the formulation leaves the device, reaches the patient, and ultimately deposits within the intended pulmonary compartment. These parameters become particularly important when comparing devices or translating a formulation from preclinical testing to human use [23, 50].

Device‐associated stress can also alter nanocarrier integrity. Nebulization exposes formulations to shear and air–liquid interfaces that may promote aggregation, leakage, or loss of nucleic‐acid activity, whereas dry powders must preserve carrier structure during drying, storage, and redispersion [29, 60]. Compatibility with pulmonary fluids after deposition adds a further constraint because surfactant and airway secretions can modify carrier dispersion and biological performance [50]. Device selection should therefore be treated as part of nanomedicine design rather than as a downstream development decision.

### 6.3 Safety and Toxicology

Safety remains a central consideration for inhaled nanomedicine because inhaled particles interact directly with the respiratory epithelium and immune system. While many nanocarriers are composed of biocompatible materials, their biological behavior may differ substantially from that of the free drug.

Potential concerns include pulmonary inflammation, oxidative stress, epithelial injury, macrophage activation, and unintended immune responses [34]. The emergence of inhaled nucleic acid delivery systems has introduced additional considerations related to immunogenicity, repeated dosing, and long‐term biodistribution. In some preclinical studies, pulmonary LNP administration has been associated with acute inflammatory responses, highlighting the importance of balancing delivery efficiency with tolerability [29].

Safety assessment is further complicated by the need to evaluate both the therapeutic cargo and the carrier platform. Long‐term studies examining repeated administration, chronic retention, and potential effects on lung function remain essential for technologies intended for chronic respiratory diseases [141]. Repeated‐dose toxicology should therefore evaluate pulmonary inflammation, histopathology, lung function, systemic biodistribution, carrier clearance, and recovery after treatment rather than relying on acute tolerability alone.

### 6.4 Regulatory Landscape

Regulatory evaluation of inhaled nanomedicines presents unique challenges because these products combine characteristics of drug formulations, delivery devices, and advanced therapeutic platforms. Regulatory agencies such as the U.S. Food and Drug Administration (FDA) and the European Medicines Agency (EMA) generally evaluate inhaled nanomedicines using existing pharmaceutical frameworks while requiring additional characterization of nanomaterial properties [144]. The FDA further recommends product‐specific evaluation of critical nanomaterial attributes, including particle‐size distribution, physical stability, manufacturing controls, and their potential effects on product quality, safety, and biological performance [145]. The regulatory emphasis is not identical across regions. The FDA applies a product‐specific, risk‐based assessment of nanomaterial attributes within the existing drug‐development framework [145], whereas EMA guidance for inhalation products places additional emphasis on formulation‐device performance, delivered‐dose uniformity, and aerodynamic particle‐size distribution [146].

Regulators increasingly emphasize detailed physicochemical characterization, manufacturing consistency, aerosol performance, biodistribution, and safety profiling [140, 141]. Particular attention is often directed toward demonstrating comparability across production batches and establishing clear relationships between formulation attributes and clinical performance.

The approval of liposomal amikacin inhalation suspension represented an important regulatory milestone because it demonstrated that complex inhaled nanomedicines can successfully navigate the clinical and regulatory pathway [45]. However, the diversity of emerging platforms, including EVs, biomimetic nanoparticles, and gene‐delivery systems, continues to raise new regulatory questions that will likely evolve alongside technological innovation.

### 6.5 Preclinical and Clinical Evidence

Clinical translation has accelerated in recent years, although the number of approved inhaled nanomedicines remains limited (Table 4). To date, the strongest clinical evidence comes from infectious lung diseases, where liposomal amikacin demonstrated improved sputum culture conversion rates in patients with treatment‐refractory *Mycobacterium avium* complex lung disease and ultimately received regulatory approval [45, 147].

#### 6.5.1 Preclinical Evidence

Preclinical in vivo studies provide the principal evidence base for many emerging inhaled nanomedicine platforms that have not yet entered clinical development. Across pulmonary fibrosis, inflammatory lung injury, cancer, and nucleic‐acid delivery, these studies have evaluated whether localized nanocarrier administration can improve pulmonary retention, enhance target‐cell exposure, modulate disease‐associated pathways, and reduce systemic exposure. However, therapeutic efficacy remains strongly dependent on disease model, target‐cell accessibility, carrier composition, and the biological barrier being addressed. The preclinical evidence therefore supports a pathway‐ and target‐driven development strategy rather than direct extrapolation of pulmonary accumulation to clinical efficacy.

#### 6.5.2 Clinical Trials of Inhaled Nanomedicines

Beyond approved products, numerous clinical and early‐phase studies have evaluated liposomal antibiotics, inhaled chemotherapeutics, nanoparticle‐based anti‐inflammatory therapies, and pulmonary gene‐delivery platforms [29, 41]. However, many candidates have encountered challenges related to patient selection, disease heterogeneity, endpoint selection, and demonstrating meaningful clinical benefit.

The next generation of clinical trials is likely to focus increasingly on disease‐modifying strategies rather than symptomatic treatment. This shift is particularly evident in pulmonary fibrosis, lung cancer, and emphysema, where inhaled nanomedicines are being developed to deliver nucleic acids, regenerative therapies, and targeted biologics [33]. Success will depend not only on technological innovation but also on the design of clinical studies capable of demonstrating clear advantages over existing standards of care. Trial design should also reflect disease kinetics. In pulmonary fibrosis, studies should account for baseline disease severity and heterogeneous progression, with longitudinal lung‐function outcomes supported by imaging or biomarker measures. In critical ARDS, treatment timing, oxygenation severity, ventilation strategy, and compatibility of aerosol delivery with mechanical ventilation should be predefined, with clinically meaningful outcomes such as ventilator‐free survival or mortality prioritized over pharmacodynamic evidence alone.

Overall, these considerations illustrate that clinical translation is no longer limited by nanocarrier design alone. The future success of inhaled nanomedicines will depend on the integration of scalable manufacturing, robust safety assessment, regulatory alignment, and carefully designed clinical trials that address the unique challenges of pulmonary drug delivery.

## 7 Emerging Trends and Future Directions

The next phase of inhaled nanomedicine will depend on moving from platform development to clinically guided design (Figure 4). Future progress will require formulations that are not only stable and inhalable, but also matched to disease biology, target cell identity, patient biomarkers, and therapeutic intent. This shift is expected to connect precision medicine, biomarker‐guided delivery, artificial intelligence, biomimetic careers, regenerative strategies, and rational combination therapies into a more integrated pulmonary treatment model.

**FIGURE 4:**
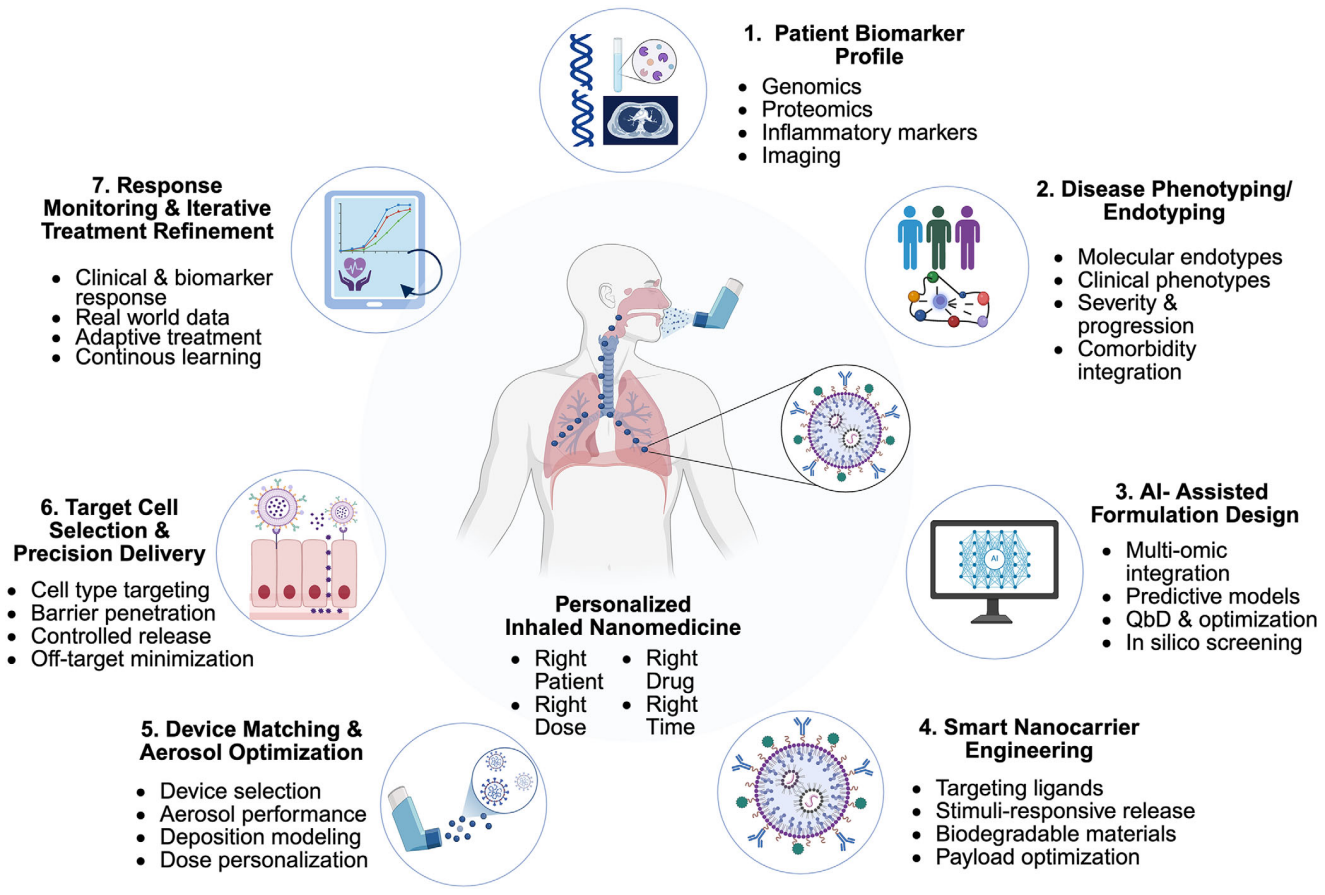
Precision framework for personalized inhaled nanomedicine. The schematic highlights how biomarker profiling, disease endotyping, AI‐guided formulation design, smart nanocarriers, optimized inhalation devices, and response monitoring can be integrated to improve targeted and individualized pulmonary therapy. Figure created using *Biorender.com*.

### 7.1 Precision Pulmonary Medicine

Precision pulmonary medicine will push inhaled nanomedicine away from one formulation that fits all treatments. Lung diseases are biologically heterogeneous. The same diagnosis can involve different epithelial, immune, endothelial, fibrotic, or tumor pathways. Future formulations should therefore be designed around target cells and disease endotypes. AT2 cell delivery may be more relevant for fibrosis and surfactant disorders (Figure 4). A macrophage‐directed system may be more useful in COPD, infection, and ARDS. Tumor‐targeted carriers may support local chemotherapy, RNA therapy, or immunotherapy in lung cancer. This cell‐specific logic is already shaping pneumonocyte‐targeted nanomedicine and broader inhaled nanoparticle development. It should also improve patient selection and translational study design [138, 148].

### 7.2 Biomarker‐Guided Therapeutics

Biomarkers will help identify which patients are more likely to benefit from inhaled nanomedicine. Blood eosinophils, FeNO, septum signatures, ct‐DNA, inflammatory proteins, and genetic variants can define treatable traits more clearly than diagnosis alone [149] [150]. This is important because each lung environment may need a different delivery strategy. A fibrotic lung may require antifibrotic cargo. An eosinophilic airway may require immune modulation, and a tumor‐rich lung may require local cytotoxic or RNA‐based therapy. Biomarker‐guided design can also reduce unnecessary exposure during early clinical testing.

### 7.3 Artificial Intelligence in Nanomedicine Design

Artificial intelligence may reduce empirical screening by linking lipid structure and formulation variables with delivery performance. Recent AI‐assisted LNP platforms have already demonstrated high‐throughput formulation optimization [151]. More specifically, Wang et al. used AI‐guided virtual screening to evaluate nearly 20 million candidate ionizable lipids and experimentally validated several high‐performing structures for mRNA delivery [152]. Computational tools can also support aerosol optimization. Islam et al. combined machine learning with CFD‐DEM simulations to predict pharmaceutical aerosol deposition across different particle sizes and airflow conditions without repeating computationally intensive simulations [153]. Together, these approaches suggest that future inhaled nanomedicine development could integrate molecular screening with predicted aerosol behavior before experimental formulation testing.

### 7.4 Biometric and Cell ‐Derived Nanocarriers

Biomimetic nanocarriers are attractive because they borrow useful features from natural cells and vesicles. Cell membrane‐coated nanoparticles can carry surface proteins that support immune evasion, adhesion, or inflammatory targeting. EV‐inspired systems may also improve biological communication between cells (Figure 4). In lung disease, these platforms may help target macrophages, tumors, injured endothelium, or regenerating epithelium. Their main challenge is manufacturing. Batch consistency, cargo control, sterility, storage, and safety testing must improve before this system can move confidently into pulmonary clinical use.

### 7.5 Regenerative Strategies in Inhaled Nanomedicine

Regenerative pulmonary nanomedicines aim to repair lung injury rather than only suppress inflammation. This direction is important for fibrosis, ARDS, radiation injury, and transplant‐related lung damage. MSC‐derived EV have shown repair‐oriented effects in experimental lung injury. They can reduce inflammation, vascular leakage, oxidative stress, and tissue damage [154, 155]. Future nanocarriers may deliver mRNA, microRNA, growth factors, or small molecules that support AT2 cell function and epithelial barrier recovery. This could help guide alveolar repairs after severe injury.

### 7.6 Combination Therapeutics

Future inhaled therapies may combine more than one mechanism in the same nanocarrier (Figure 4). An antifibrotic drug could be paired with siRNA. An antibiotic could be combined with antibiofilm cargo. Chemotherapy could be delivered with immunomodulatory RNA. This strategy is attractive because lung diseases often involve more than one biological process. Inflammation, infection, remodeling, and immune escape may occur together [124, 133]. The main challenge will be controlling dose balance, release timing, local toxicity, and safety during repeated inhalation.

The unresolved challenge is therefore not the absence of new carrier technologies, but their integration into a reproducible development pathway. A next‐generation inhaled nanomedicine should begin with disease endotype and target‐cell selection, followed by cargo and carrier design, computational or experimental screening, aerosol‐device optimization, disease‐relevant biological testing, and early assessment of manufacturability and repeat‐dose safety. Progress will depend on connecting these stages rather than optimizing each independently, particularly when disease heterogeneity, aerosol performance, intracellular delivery, and scalable manufacturing impose competing design requirements.

## 8 Conclusions and Prospects

Despite major advances in respiratory medicine, most current pulmonary therapies remain focused on symptom control, inflammation suppression, or slowing disease progression rather than reversing the underlying pathology. This limitation is particularly evident in diseases such as pulmonary fibrosis, emphysema, lung cancer, and chronic pulmonary infections, where treatment efficacy is often constrained by inadequate drug delivery, systemic toxicity, and the inability to target disease‐driving cellular mechanisms.

Nanomedicine has emerged as a promising platform for overcoming many of these challenges. By improving local drug exposure, enhancing cellular targeting, and enabling delivery of complex therapeutic cargo, inhaled nanocarriers have expanded the scope of what can be achieved through pulmonary administration. Beyond conventional small molecules, nanomedicines are increasingly being used to deliver biologics, nucleic acids, and regenerative therapies directly to diseased lung tissue. The growing success of inhaled LNPs, biomimetic carriers, and EV‐inspired systems highlights the rapid evolution of the field from drug delivery toward disease modification. Particularly exciting is the emergence of inhaled genetic medicines, including mRNA, siRNA, and CRISPR‐based platforms. These technologies offer the potential to modulate pathogenic pathways, restore impaired cellular function, and, in some cases, address the underlying causes of disease rather than their downstream consequences. Such approaches may prove especially important for conditions where tissue regeneration and long‐term disease modification remain unmet clinical needs. However, these technologies are not equally mature for pulmonary translation. Liposomal drug delivery has established the clearest clinical precedent, whereas targeted LNPs, biomimetic systems, and pulmonary genome‐editing approaches remain largely preclinical or at early stages of development. Their future value will therefore depend not only on therapeutic potency but also on whether durable biological effects can be achieved with acceptable delivery burden and safety.

Despite this progress, the major translational bottleneck is no longer nanocarrier development alone. Aerodynamic performance, regional lung deposition, target‐cell access, repeat‐dose tolerability, manufacturing reproducibility, and regulatory requirements must be optimized together. Clinical experience also shows that favorable pulmonary exposure or promising animal efficacy does not necessarily translate into therapeutic benefit when disease heterogeneity, formulation‐device interactions, or human immune responses are insufficiently considered. These constraints should therefore be incorporated early in formulation development rather than addressed only after efficacy has been established.

Future development should begin with the disease endotype and the pulmonary cell population that must be reached, followed by selection of the therapeutic cargo, carrier architecture, and aerosol device appropriate for that biological context. Disease‐relevant organoids and multicellular models, computational formulation screening, aerosol simulation, biomarker‐guided patient selection, and clinically relevant repeat‐dose testing could progressively reduce uncertainty before advanced development. The future impact of inhaled nanomedicine will therefore depend less on increasing carrier complexity and more on integrating biological targeting, aerosol performance, manufacturability, safety, and clinical evidence into reproducible disease‐matched therapeutic systems.

## Author Contributions

K.K., S.G., and J.S., contributed to the writing and development of the manuscript. K.K. and J.S. contributed to the preparation of figures and tables. J.S. supervised the project and performed critical revision and editing of the manuscript. All authors reviewed and approved the final version of the manuscript. All authors take complete responsibility for the integrity of the work done in this manuscript.

## Funding Statement

The authors have nothing to report.

## Conflicts of Interest

The authors declare no conflicts of interest.

## Ethics Approval

The authors have nothing to report.

## Supporting information




**Supporting File**: mco271047‐sup‐0001‐SuppMat.docx

## Data Availability

Data sharing not applicable—no new data generated, or the article describes entirely theoretical research.
