# Supplementary material for: Inhaled Nanomedicines for Lung Diseases and Injuries: Pharmacological Advances and Clinical Translation
Source: MedComm (2020). 2026 Nov 4;7(11):e71047. doi: 10.1002/mco2.71047 (PMC13733363; doi:10.1002/mco2.71047)
Supplement: Supplementary file 1 [file MCO2-7-e71047-s001.docx]

**Supporting Information**

**Inhaled Nanomedicines for Lung Diseases and Injuries: Pharmacological Advances and Clinical Translation**

*Kajal Kamra^1^, Sonal Gaur^2^, Jaswinder Singh^3*^*

**Affiliations:**

1 Department of Pharmaceutical Sciences, University of Nebraska Medical Center, Omaha, NE 68198, USA; ORCID ID: 0000-0002-6735-3826; [kajal.kamra@unmc.edu](mailto:kajal.kamra@unmc.edu)

2 Department of Ophthalmology, Medical University of South Carolina, Charleston, SC 29425, USA; ORCID ID: 0009-0009-7959-780X; [gaurs@musc.edu](mailto:gaurs@musc.edu)

3 Department of Emergency Medicine, University of Nebraska Medical Center, Omaha, NE 68198, USA; ORCID ID: 0000-0002-6004-7113; [jsingh@unmc.edu](mailto:jsingh@unmc.edu)

***Corresponding Author**

**Jaswinder Singh, Ph.D.**

University of Nebraska Medical Center,

Omaha, NE 68198, USA

e-mail: [**jsingh@unmc.edu**](mailto:jsingh@unmc.edu)

**List of Supplementary Tables**

**Table S1: Positioning of the Present Review Relative to Recent Reviews of Inhaled Nanomedicine**

| **Review** | **Principal scope** | **Biological barriers and carrier classes** | **Disease and target-cell specificity** | **Aerosol and device engineering** | **Nucleic-acid and regenerative strategies** | **Clinical, manufacturing and regulatory translation** | **Distinctive emphasis relative to the present review** |
| --- | --- | --- | --- | --- | --- | --- | --- |
| Jin et al., 2023^5^ | Pulmonary barriers and rational design of inhaled nanoparticles | Broad pulmonary barriers and established nanocarrier classes | Multiple respiratory diseases; target-cell comparison is selective | Formulation and deposition considerations; device integration is not the primary focus | Selected nucleic-acid applications; regenerative strategies are not central | Translational considerations discussed selectively; manufacturing and regulatory pathways are not the organizing framework | Emphasizes barrier-overcoming nanocarrier design |
| Zhang et al., 2023^124^ | Inhaled RNA therapeutics and nano-bio interactions | Mucus, surfactant, cellular uptake, intracellular trafficking and RNA carriers | Strong disease-related cell and target-cell orientation | Nebulization and pulmonary delivery considerations; device selection is secondary | Extensive RNA-delivery coverage; regenerative applications are not a central organizing theme | Safety and translational barriers are discussed; manufacturing and regulatory staging are selective | Connects disease-related lung cells with RNA-delivery requirements |
| Woodward & Fromen, 2024^23^ | Emerging aerosol technologies, complex cargos and pulmonary targets | Selected barriers and broad particle-engineering approaches | Regional targeting, patient heterogeneity and diverse pulmonary applications | Extensive aerosol, particle and inhaler-device engineering | Complex cargos, including biologics and nucleic acids; regeneration is not the primary focus | Strong clinical and patient-oriented perspective; manufacturing and regulatory analysis is selective | Emphasizes aerosol engineering, device innovation and personalized inhalation |
| Costabile et al., 2024^50^ | Design and development of inhalable lipid and polymer nanocarriers | Extensive lipid/polymer carrier coverage and lung-fluid interactions | Multiple diseases; target-cell comparison is selective | Strong formulation-aerosol-device considerations | Includes gene-delivery applications; regenerative strategies are not central | Development and scale-up considerations are discussed; clinical and regulatory staging is selective | Emphasizes interplay among payload, carrier composition and aerosol performance |
| Fan et al., 2025^156^ | Inhaled nanoparticle materials, dosage forms and pulmonary applications | Broad carrier classes and lung-barrier penetration | Broad disease applications; target-cell analysis is selective | Dosage-form and inhalation considerations are included | Selected advanced cargos; nucleic-acid and regenerative strategies are not comprehensively differentiated | In vitro, in vivo and clinical evidence is summarized; manufacturing/regulatory staging is not the central framework | Provides broad platform- and disease-oriented coverage |
| Zhang et al., 2026^157^ | Inhalable materials and biologics for lung defence and drug delivery | Extensive mucus-barrier analysis with selected materials and biologics | Infectious and chronic pulmonary diseases; primarily focused on the mucus interface | Pulmonary delivery strategies are discussed; inhaler-device comparison is not central | Selected biologics and advanced cargos; genetic and regenerative modalities are not comprehensively compared | Clinical implications are discussed; manufacturing and regulatory pathways are not the primary focus | Examines the balance between mucus-mediated defence and therapeutic delivery |
| Present review | Disease endotype → target cell → cargo → carrier → device → evidence level → clinical stage | Integrates anatomical, mucus, surfactant, epithelial, immune, inflammatory and disease-specific barriers with major carrier classes | Compares fibrosis, lung cancer, COPD/emphysema, pulmonary infections and ALI/ARDS according to relevant target cells | Connects carrier and cargo selection with aerosol stability, regional deposition and device requirements | Differentiates mRNA replacement, siRNA silencing, ASO-mediated splicing, CRISPR nuclease editing, base editing, prime editing and regenerative strategies | Integrates approved products, clinical trials, evidence maturity, safety, manufacturing, device compatibility and regulatory considerations | Provides an end-to-end framework linking disease biology with delivery design and clinical-development readiness |

**Table S2: Pulmonary Delivery Barriers, Disease Context, and Nanomedicine Design Implications**

| **Pulmonary barrier** | **Consequence for delivery** | **Disease context** | **Design implication** | **Representative evidence** |
| --- | --- | --- | --- | --- |
| **Aerodynamic deposition constraints** | Limits distal deposition and increases extra thoracic loss | All inhaled indications; particularly heterogeneous obstructive disease | Match aerodynamic properties and device performance to the intended lung compartment; nano-in-microparticle or porous systems can separate aerosol deposition from post-deposition nanoscale delivery | **^23-25^** |
| **Mucus barrier** | Traps particles and accelerates mucociliary removal | CF, COPD, bronchiectasis, chronic bronchitis | Minimize adhesive interactions; dense hydrophilic coatings can improve transport through pathological mucus | **^26,27^** |
| **Pulmonary surfactant** | Alters dispersion, aggregation and cellular interaction | Distal-lung and alveolar delivery | Evaluate nanocarrier stability and biological activity in surfactant-containing conditions rather than relying on conventional cell culture alone | **^28,29^** |
| **Respiratory epithelium and endosomal trafficking** | Restricts productive intracellular delivery | CF, RNA therapy, fibrosis | Optimize cellular uptake and endosomal escape according to the intracellular site of action of the cargo | **^31,62,124^** |
| **Cell-type heterogeneity** | High total lung uptake may occur in the wrong cell population | CF, fibrosis, emphysema, cancer | Define the therapeutic target cell before optimizing carrier tropism | **^32,33,85^** |
| **Alveolar macrophage clearance** | Produces non-productive particle sequestration or rapid clearance | Distal-lung delivery; RNA therapeutics | Avoid macrophage uptake when epithelial targeting is required, but exploit it when macrophages are therapeutic targets | **^29,34^** |
| **Inflammatory microenvironment** | ROS, proteases and NETs can alter carrier stability and drug release | COPD, infection, ARDS | Use inflammation-responsive chemistry only when the pathological trigger is sufficiently disease-enriched | **^35,36^** |
| **Fibrotic extracellular matrix** | Increases diffusion distance and restricts lesion penetration | IPF/fibrotic ILD | Optimize penetration and cellular targeting rather than pulmonary retention alone | **^37-40^** |
| **Tumor microenvironment** | Hypoxia, stromal barriers and heterogeneous distribution limit tumor exposure | Lung cancer | Combine local deposition with tumor-cell or microenvironment-directed delivery | **^41,42^** |
| **Biofilms and intracellular reservoirs** | Reduce antimicrobial exposure at the pathogen niche | Bronchiectasis, NTM disease, tuberculosis | Engineer biofilm penetration or macrophage-directed delivery according to pathogen localization | **^43-46^** |

**Table S3: Inhaled Nanomedicine Platforms Across the Translational Evidence Continuum**

| **Platform** | **Pulmonary target/ pathway and cargo** | **Dominant design advantage** | **Principal limitation/safety concern** | **Evidence category represented in this review** | **Representative evidence** |
| --- | --- | --- | --- | --- | --- |
| **Liposomes** | Airway infection; antibiotics and small molecules | Sustained local exposure and established pulmonary regulatory precedent | Drug leakage, formulation stability, local tolerability | Approved: ALIS/Arikayce for treatment-refractory MAC lung disease; additional liposomal formulations have undergone clinical testing | ^44,45,147^ |
| **LNPs** | Airway/alveolar epithelium; mRNA, siRNA and editing cargo; gene replacement/silencing/editing | Efficient intracellular nucleic-acid delivery | Nebulization stability, innate immune activation and non-target-cell uptake | Clinical-stage: MRT5005 CFTR mRNA; Preclinical: nebulized mRNA, epithelial siRNA and CRISPR delivery | ^29,85,125,134^ |
| **Polymeric nanoparticles** | Airway/mucus and intracellular targets; small molecules and nucleic acids | Tunable release and surface chemistry | Polymer degradation, pulmonary tolerability and scale-up | Predominantly preclinical in pulmonary applications discussed here | ^27^ ^158^ |
| **Lipid-polymer hybrid nanoparticles** | Pulmonary tumor/epithelial targets; drugs and RNA | Combines polymer stability with lipid-mediated cellular interaction | Formulation complexity and reproducibility | Preclinical | ^77^ |
| **Cell membrane-coated nanoparticles** | Inflamed lung/macrophage-directed applications; biomimetic recognition | Biomimetic recognition and inflammatory-site targeting | Source variability, membrane integrity, scale-up and immunological characterization | Preclinical | ^78^ |
| **Extracellular-vesicle systems** | Tumor, inflammatory and regenerative targets; RNA/proteins; intercellular cargo delivery | Natural intercellular delivery and biological tropism | Production yield, cargo consistency, purification and storage | Preclinical | ^81^ |
| **Stimuli-responsive systems** | ROS-, pH- or enzyme-rich lesions; drugs/RNA; trigger-dependent drug release | Trigger-dependent local release | Trigger heterogeneity, formulation complexity and unpredictable release in humans | Preclinical / proof-of-concept | ^101^ |
